# Two-Step Regulation of a Meristematic Cell Population Acting in Shoot Branching in *Arabidopsis*

**DOI:** 10.1371/journal.pgen.1006168

**Published:** 2016-07-11

**Authors:** Bihai Shi, Cui Zhang, Caihuan Tian, Jin Wang, Quan Wang, Tengfei Xu, Yan Xu, Carolyn Ohno, Robert Sablowski, Marcus G. Heisler, Klaus Theres, Ying Wang, Yuling Jiao

**Affiliations:** 1 State Key Laboratory of Plant Genomics, Institute of Genetics and Developmental Biology, Chinese Academy of Sciences, and National Center for Plant Gene Research, Beijing, China; 2 University of Chinese Academy of Sciences, Beijing, China; 3 Department of Plant Breeding and Genetics, Max Planck Institute for Plant Breeding Research, Cologne, Germany; 4 Agricultural Genomics Institute at Shenzhen, Chinese Academy of Agricultural Sciences, Shenzhen, China; 5 State Key Laboratory of Crop Stress Biology in Arid Areas, Ministry of Agriculture Key Laboratory of Horticultural Plant Biology and Germplasm Innovation in Northwest China, College of Horticulture, Northwest A&F University, Yangling, Shaanxi, China; 6 Developmental Biology Unit, European Molecular Biology Laboratory, Heidelberg, Germany; 7 Cell and Developmental Biology Department, John Innes Centre, Norwich Research Park, Norwich, United Kingdom; 8 Frontier Laboratory of Crop Design, Beijing, China; The University of North Carolina at Chapel Hill, UNITED STATES

## Abstract

Shoot branching requires the establishment of new meristems harboring stem cells; this phenomenon raises questions about the precise regulation of meristematic fate. In seed plants, these new meristems initiate in leaf axils to enable lateral shoot branching. Using live-cell imaging of leaf axil cells, we show that the initiation of axillary meristems requires a meristematic cell population continuously expressing the meristem marker *SHOOT MERISTEMLESS* (*STM*). The maintenance of *STM* expression depends on the leaf axil auxin minimum. Ectopic expression of *STM* is insufficient to activate axillary buds formation from plants that have lost leaf axil *STM* expressing cells. This suggests that some cells undergo irreversible commitment to a developmental fate. In more mature leaves, *REVOLUTA* (*REV*) directly up-regulates *STM* expression in leaf axil meristematic cells, but not in differentiated cells, to establish axillary meristems. Cell type-specific binding of REV to the *STM* region correlates with epigenetic modifications. Our data favor a threshold model for axillary meristem initiation, in which low levels of *STM* maintain meristematic competence and high levels of *STM* lead to meristem initiation.

## Author Summary

In seed plants, branches arise from axillary meristems (AMs), which form in the crook between the leaf and the stem. How AMs initiate to produce branches remains unclear. In this study, we show that a group of meristematic cells maintain expression of the meristem marker *SHOOT MERISTEMLESS* (*STM*); the progeny of these cells form the axillary buds. Our results suggest that low-level *STM* expression is required (but not sufficient) for AM initiation, and that high-level *STM* expression induces initiation of the AM. The initial expression of *STM* requires the auxin minimum in the leaf axil and the transcription factor REVOLUTA directly up-regulates *STM* expression.

## Introduction

In plants, many somatic cells can regenerate into complete plants; thus, many plant cells are considered totipotent, unlike most somatic cells in animals [1]. Plants also show well-defined developmental patterning, which leads to questions about how cell fates become established. Specialized cell lineages generate guard cells or pavement cells in the leaf epidermis [2], and produce callus during regeneration [3]; both of these cell types have similarities to animal stem cell lineages. Much less is known about cell fate determination in other aspects of plant development.

An iconic feature of plants is their branching growth habit, an innovation considered crucial for their conquest of land [4, 5]. Plants maintain meristems with undifferentiated stem cells, which are responsible for the life-long organogenesis of growing plants. Branching occurs by periodic initiation of new meristems. In the seed plants, secondary growth axes arise from axillary meristems (AMs, also termed lateral meristems) in or near the adaxial side of leaf axils [6–8]. During AM initiation, a morphologically detectable bump forms in the leaf axil and develops into a bud [9–11]. Two models have been proposed to explain AM initiation. The ‘detached meristem’ model proposes that a few pluripotent cells detach from the primary shoot apical meristem (SAM) and associate with the leaf axil as the leaf differentiates from the SAM [10, 12]. Histological analysis shows that leaf axil cells likely remain undifferentiated, providing support for the detached meristem theory [12, 13]. Analysis of the *Arabidopsis thaliana phabulosa-1d* mutant led to the alternative ‘*de novo* induction’ model [14], in which an AM initiates from differentiated leaf cells. A major difference between these models is whether AM initiation requires a meristematic cell lineage [10, 15, 16].

Although the origin of AMs is presently unclear, genetic studies in *Arabidopsis* have shown that AM initiation is regulated by several transcription factor-encoding genes, such as *LATERAL SUPPRESSOR* (*LAS*), *REGULATOR OF AXILLARY MERISTEMS*, *CUP-SHAPED COTYLEDON* (*CUC*), and *REGULATOR OF AXILLARY MERISTEM FORMATION* [11, 17–20]. Genetic and molecular studies revealed direct and indirect interactions among these genes in a regulatory network [18, 21]. Many of these genes have conserved functions in the regulation of AM initiation in dicots and monocots, such as tomato (*Solanum lycopersicum*), maize (*Zea mays*), and rice (*Oryza sativa*) [22–26]. Phytohormones also regulate AM initiation, which requires an auxin minimum and a subsequent cytokinin signaling pulse [9, 27, 28].

Here, we asked whether post-embryonic AM initiation requires meristematic cells with a fixed developmental fate [10, 11, 15], and how these cells are regulated. Our results show that initiation of branching meristems in the shoot requires a meristematic cell population embedded in differentiated cells. Examination of the fine-tuning of these cells led to a threshold model for AM initiation.

## Results

### A Meristematic Cell Population in Leaf Axils

Previous *in situ* hybridization results showed *STM* expression in all stage leaf axils from examination of fixed samples, but it remains unclear if a continuous *STM*-expressing cell population exists during development [10, 11, 16]. The *STM*-expressing cells are closer to the meristem side of the boundary in young leaf primordia, and are closer to the leaf side of the boundary in older leaves. Thus, it has been proposed that the initial *STM*-expressing cells may create a separation while their neighboring cells re-differentiate as AM progenitor cells [10]. To better resolve the origin of *STM* expressing cells, we used live-cell imaging to determine if a continuous *STM*-expressing cell population exists in the leaf axil. To this end, we imaged axils of living leaf primordia that we isolated from the shoot apex and maintained in culture. As shown previously, cultured leaf primordia (P_6_ and older) efficiently initiated AMs in the absence of exogenous phytohormone [9], which is distinct from *de novo* organogenesis [29].

**Fig 1:**
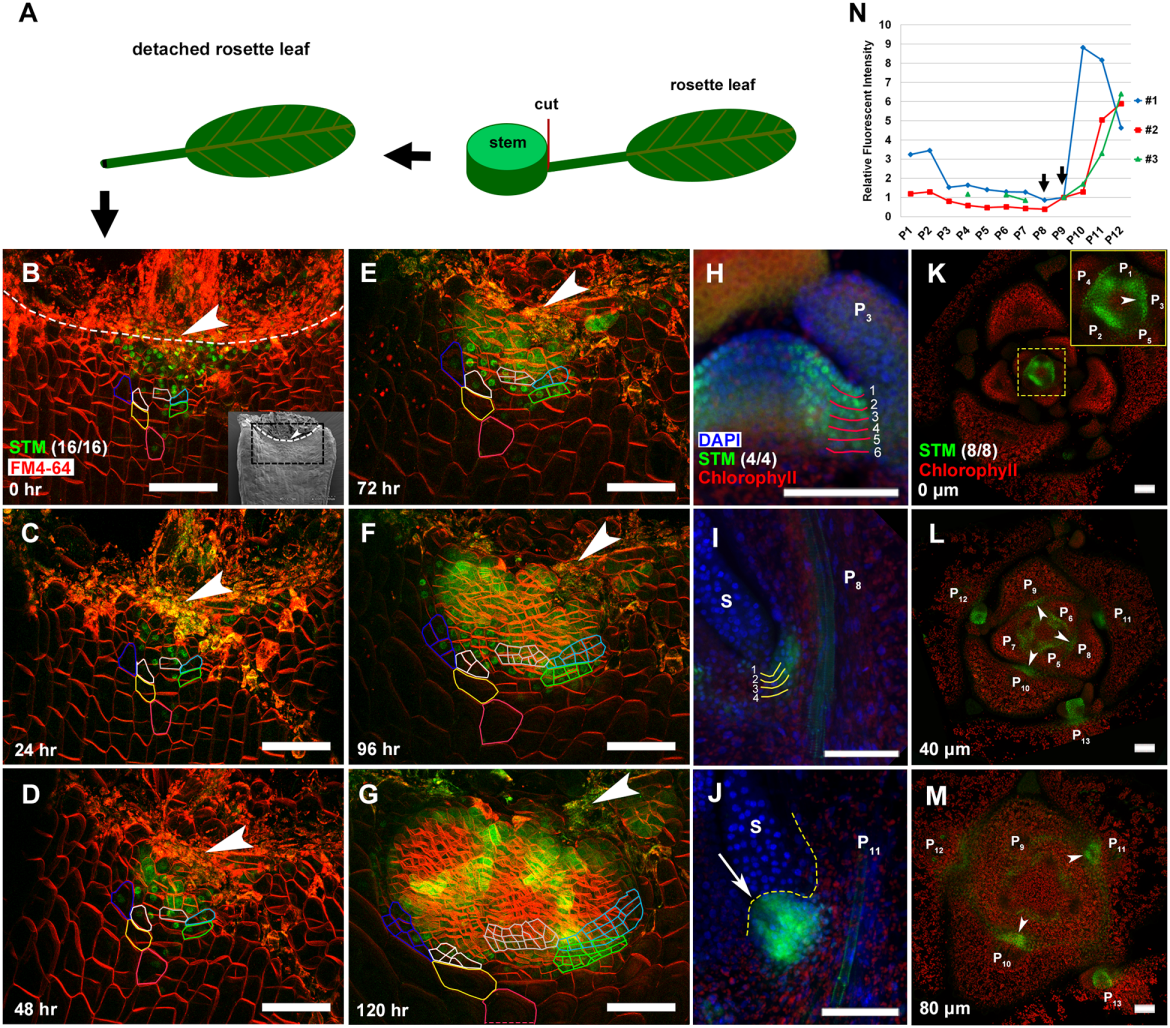
Existence of a meristematic cell population with a fixed developmental fate in leaf axils. (A) Schematic flow showing isolation of a rosette leaf primordium for AM live imaging. The black square at the end of the petiole disproportionately highlights the region of imaging. (B-G) Reconstructed view of the L1 layer of a P_9_ leaf axil with STM-Venus (green) expression and FM4-64 stain (red) showing location and lineage of AM progenitor cells, with (B) being the first time point and elapsed time in (C-G). Selected progenitor cells are color-coded, and the same color has been used for each progenitor cell and its descendants. The white line indicates the incision line at the leaf axil. Arrowheads highlight the approximate center of the incision line. Insert in (B) shows scanning electron micrograph of a rosette leaf axil of similar stage. The box bordered by the black dotted line roughly corresponds to the region imaged by confocal microscopy, and the white dotted lines marks incision line. (H-J) Longitudinal sections through *pSTM*::*STM-Venus* leaf axils of vegetative SAMs stained with DAPI (blue) showing a *STM*-expressing cell population. STM-Venus (green) was initially expressed in 6 files of cells at stage P_3_ (H), later decreased to 4 files at P_8_ (I), and then expanded to more cells at P_11_ prior to AM initiation (J). The arrow in (J) highlights the bulged meristem. Note the expression of *STM* was substantially higher in the boundary than in the SAM, as shown before [30]. (K-M) Continuous transverse sections through a vegetative shoot apex, showing expression of STM-Venus (green) in leaf axils (arrowheads). Sections are ordered from most apical (K) to most basal (M); approximate distance (in micrometers) from the summit of the SAM to section is given in the bottom left-hand corner of each image. Note a significant increase in STM-Venus signal between P_9_ and P_10_ (L and M). (N) Relative fluorescence intensity of STM-Venus at leaf axils from P_1_ to P_12_. 3 replications were done by analysis the transverse sections like in (K-M). Arrows highlight the lowest value at P_8/9_. Bars = 50 μm.

By live-imaging the expression of a functional *pSTM*::*STM-Venus* reporter in P_6_ and older leaves (Fig 1A), we found that cells with continuous STM-Venus expression are AM progenitors. A recent study has shown that this reporter line can fully complement *stm* mutation, and the enhanced boundary expression reflects the endogenous *STM* expression [30]. For the P_8/9_ leaf primordium, which has the fewest *STM*-expressing cells of all the stages (see below), we observed the STM-Venus signal only in a continuous cell mass close to the incision line (Fig 1B). The number of cells with STM-Venus signal initially decreased after 24 h of culture (Fig 1C), but partially recovered after 48 h of culture (Fig 1D). Starting from 72 h of culture, following a series of rapid cell divisions (Fig 1E), these cells organized into a meristem with new leaf primordia (Fig 1F and 1G). Occasionally, a few cells without initial STM-Venus signal at the first time point, but next to one or more STM-positive cells, showed detectable signal. These cells could have initially had low-level STM-signals below our detection threshold. Alternatively, their *STM* expression could be due to STM proteins trafficked from neighboring *STM*-expressing cells [31].

In early stage leaf axils, STM-Venus also persisted in the boundary region. In tissue sections, the number of *STM*-expressing cells gradually decreased during leaf primordia maturation from P_3_ (Fig 1H) to P_9_ (Fig 1I). Later, in P_10_ and older leaves, the number of *STM*-expressing cells and level of *STM* expression increased significantly (Fig 1J), which is consistent with the live-imaging results. Quantitative measurement of leaf axil STM-Venus fluorescence intensity confirmed this *STM* expression dynamic pattern during leaf maturation. In particular, P_8_ or P_9_ have the lowest intensity in L*er*, which significantly increase in the next developmental stage (Fig 1K–1N and S1A–S1C Fig). There is a small variation between individual plants with either P_8_ or P_9_ having the lowest *STM* expression, which is in line with a previous morphological analysis (10).

In addition, we also used reporter lines to follow expression of the shoot meristem central zone stem cell marker *CLAVATA3* (*CLV3*), the shoot meristem organizing center marker *WUSCHEL* (*WUS*), and the pericycle-like cell marker J0121, which marks progenitor cells for regeneration [3, 32, 33]. We did not detect *CLV3* or *WUS* expression in young L*er* leaf axils until the twelfth-youngest primordium (P_12_, S1D–S1F Fig) and J0121 was not expressed at all in the leaf axil during axillary bud formation (S1G Fig), suggesting that their corresponding cell identities were not maintained.

### STM-expressing Cells Are Required for AM Initiation

**Fig 2:**
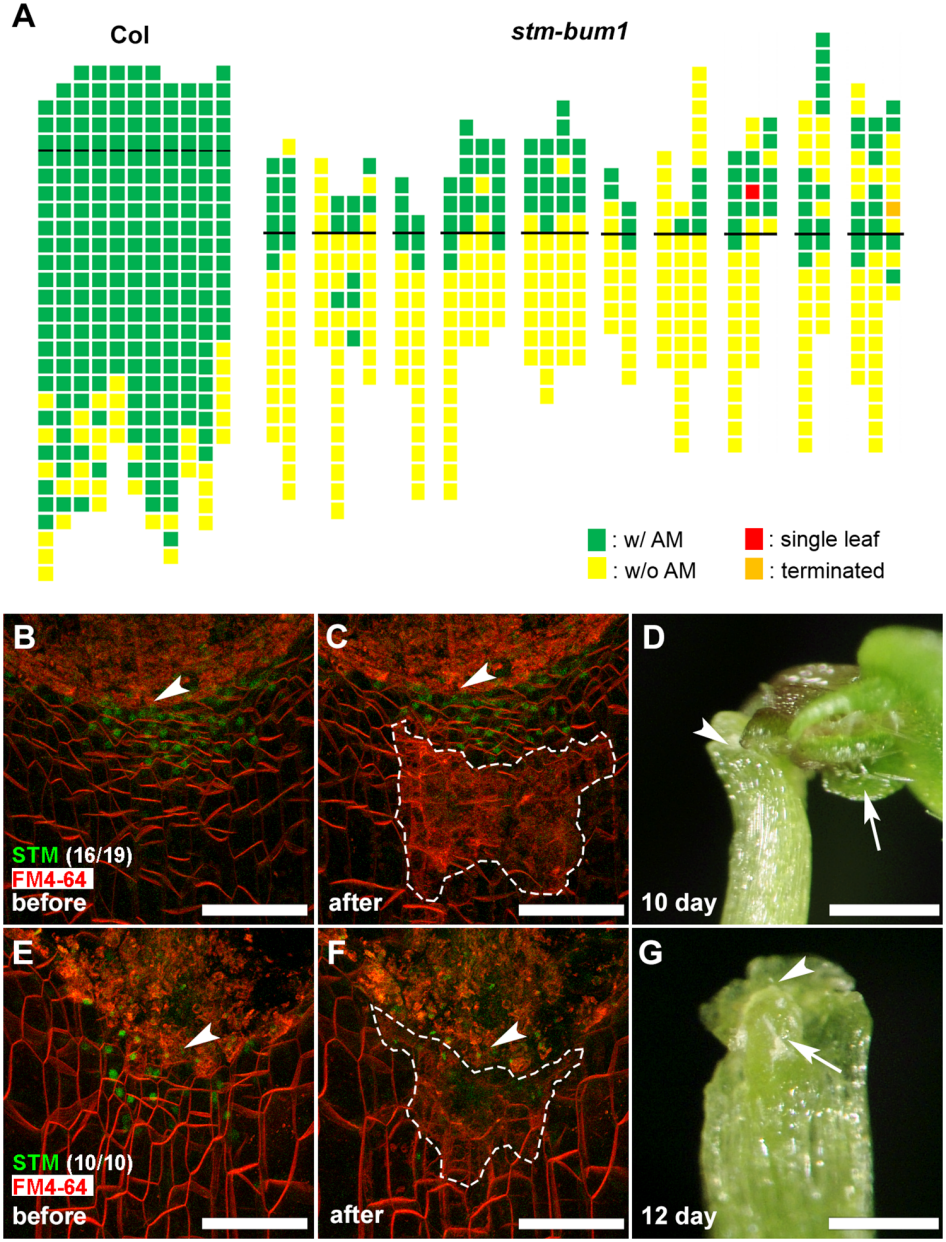
AM initiation requires *STM*-expressing cells. (A) Schematic representation of axillary bud formation in leaf axils of Col-0 wild-type plants and the *stm-bum1* mutant plants. The thick black horizontal line represents the border between the youngest rosette leaf and the oldest cauline leaf. For Col-0, each column represents a single plant, and each square within a column represents an individual leaf axil. For *stm-bum1*, each column represents a single main branch, and branches from a single plant are grouped together. The bottom row represents the oldest rosette leaf axils, with progressively younger leaves above. Green indicates the presence of an axillary bud, yellow indicates the absence of an axillary bud, red indicates the presence of a single leaf in place of an axillary bud, and orange indicates the presence of a terminated axillary bud in any particular leaf axil. (B, C, E and F) Leaf axils (as shown in Fig 1B) showing cells with STM-Venus (green) expression before laser ablation (B and E) and after laser ablation (C and F). The regions bordered by the white dotted line (in C and F) are subject to laser ablation. (D and G) Axillary bud formation 12 d after laser ablation, as shown in (C and F). Arrows show presence (D) and absence (G) of an axillary bud. Note axillary buds formed only when *STM*-expressing cells remain intact (B-D). Arrowheads highlight the approximate center of the incision lines. Bars = 50 μm in (B, C, E, and F) and 1 mm in (D and G).

**Fig 3:**
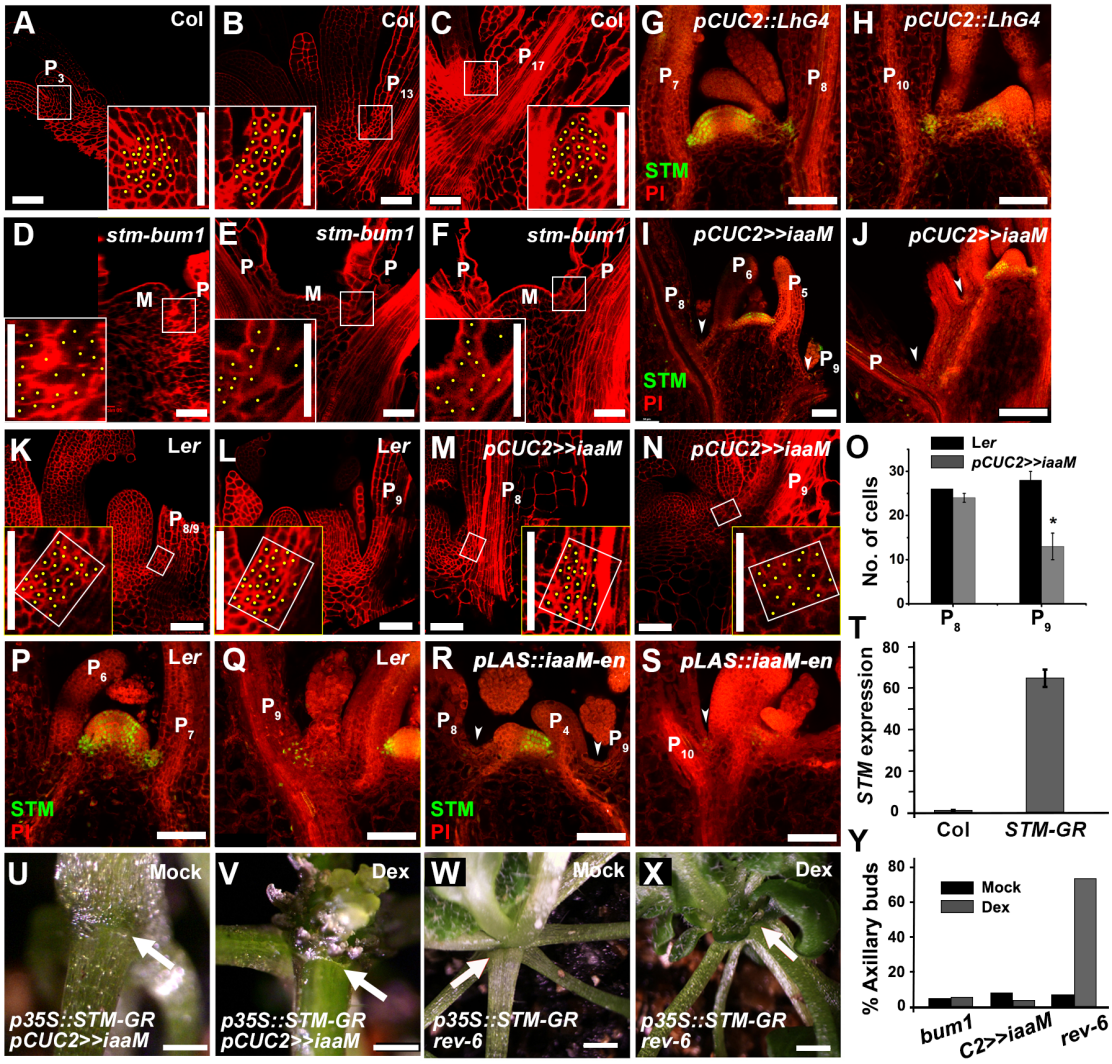
Low level *STM* expression is required for meristematic cell identity and axillary buds formation. (A-F) Optical longitudinal sections through the middle of leaf axils of vegetative SAMs stained with mPS-PI showing cell morphology in 30-day-old wild type (A-C) and in *stm-bum1* (D-F) plants. The inserts show corresponding magnified leaf axil region indicated by white boxes. Each cell receives a yellow dot. Note the enlarged cells in *stm-bum1*. Because *stm-bum1* meristems terminate after producing a small number of leaves, leaves (P) of comparable size to the wild type were chosen. M represents the vegetative meristem. (G-J) Expression of STM in leaf axils of *pCUC2*::*LhG4* (G and H) and *pCUC2>>iaaM* (I-J) plants. Longitudinal sections through vegetative shoot apexes stained with propidium iodide (PI, red) showing expression of *pSTM*::*STM-Venus* (green). Arrowheads highlight leaf axils. Note that the STM-Venus signal diminished in older ones. P in (J) marks an undetermined old leaf. (K-N) Optical longitudinal sections through the middle of leaf axils of vegetative SAMs stained with mPS-PI showing cell morphology in 30-day-old wild type (K and L) and in *pCUC2>>iaaM* (M and N) plants. The inserts show corresponding magnified leaf axil region indicated by white boxes. Note leaf axil cells in P_9_ of *pCUC2>>iaaM* are enlarged as in *stm-bum1*. Each leaf axil cell received a yellow dot. (O) Statistical analysis of number of cells at leaf axils corresponding to the rectangle shown in (K-N). Note the distinguished difference between P_9_ of L*er* and of *pCUC2>>iaaM (P* < 0.05). (N-Q) Expression of STM in leaf axils of L*er* (P and Q) and *pLAS*::*IAAM-en* (R and S) plants. Arrows in (R and S) indicate leaf axils. (T) RT-qPCR assay of *STM* expression in leaf removed shoot apex tissues of Col-0 and *p35S*::*STM-GR* plants. Bars = 50 μm in (A-N and P-S), 500 μm in (U and V) and 1 mm in (W and X). (U-V) Close-up of rosette leaf axils in *p35S*::*STM-GR pCUC2>>iaaM* after two continuous days mock treatment (U) and Dex treatment (V). Arrows indicate absence of an axillary bud. (W-X) Rescue of AM defect in *rev-6* by inducible STM-GR activation. Close-up of rosette leaf axils in *p35S*::*STM-GR rev-6* after one time mock treatment (W) and Dex treatment (X), showing absence and presence (arrows) of an axillary bud, respectively. (Y) Graphic representations of axillary bud formation after mock or Dex induction of *p35S*::*STM-GR* during vegetative development in corresponding genotypes. Twelve-days-old plants were treated for five days. Only leaves visible before treatment were counted. The percentage values indicate the proportion of plants analyzed (n > 15) that formed at least one axillary bud.

We next tested whether AM initiation required the *STM*-expressing cells. It has been reported that mild *stm* alleles have more active branching and show an ‘abort-retry’ mode of growth [34–36]. However, because SAM termination (in *stm* mutants) promotes outgrowth of axillary buds, branch growth may not reflect axillary bud formation. To show if *STM* functions in AM initiation, we analyzed the pattern of axillary bud formation in plants carrying the weak *stm-bum1* allele, which can still form leaves from a partially functional SAM [37]. We found a dramatic reduction in the number of axillary buds in *stm-bum1* plants, with 60% (242 out of 405) of leaves lacking axillary buds, which is distinct from Col-0 wild-type plants (Fig 2A). This reduction in axillary buds is more dramatic for rosette leaves (91% leaves lack buds). In contrast to the wild type (Figs 1I and 3A–3C), leaf axil cells in *stm-bum1* plants are enlarged (Fig 3D–3F), suggesting that the leaf axil cells have undergone differentiation. On the other hand, *stm-bum1* plants have reduced apical dominance, resulting in the reported enhanced branching phenotype [34–36].

To test if AM initiation requires *STM*-expressing cells, we applied laser ablation. When we ablated the cells adjacent to the *STM*-expressing cells, AMs initiated normally from the ablated leaf axil region (Fig 2B–2D, 16 out of 19), showing that ablation *per se* does not abolish AM initiation. However, after ablation of most cells within the *STM*-expressing cell mass in both the epidermis and internal cell layers, AMs could not initiate (Fig 2E–2G, 10 out of 10). Note that this result is in contrast to observations in shoot and root apical meristems [38–41], where neighboring cell fate can switch after ablation. Furthermore, we observed that AMs did not initiate from cultured leaves if we removed the proximal portion of the petiole containing the *STM*-expressing cells (S2A–S2C Fig). Taken together, our data strongly suggest that AM initiation requires the *STM*-expressing cells as AM progenitor cells.

### Maintenance of *STM* Expression Requires the Leaf Axil Auxin Minimum

We next asked what regulates the maintenance of *STM* expression in the leaf axil. We have recently shown that the AM progenitor cells also maintained a low auxin level [9, 27], suggesting that maintenance of *STM* expression may require the leaf axil auxin minimum. To test this hypothesis, we analyzed *STM* expression in *pCUC2>>iaaM* and *pLAS*::*iaaM-en* plants, which ectopically accumulate auxin in leaf axils and are deficient in AM initiation [9, 27]. We could not detect *STM* expression in leaf axils of *pCUC2>>iaaM* plants (Fig 3G–3J). In addition, leaf axil cells in *pCUC2>>iaaM* plants are enlarged (Fig 3K–3O), suggesting cell differentiation. Similarly, *pLAS*::*iaaM-en* plants also have substantially reduced or undetectable leaf axil *STM* expression and have enlarged leaf axil cells (Fig 3P–3S).

To test if *STM* expression alone is sufficient for AM initiation, we introduced *p35S*::*STM-GR* into *pCUC2>>iaaM* plants. In *p35S*::*STM-GR* plants [42], dexamethasone (Dex) can induce the nuclear translocation of a STM-glucocorticoid-receptor (GR) fusion protein. We aimed to test if leaf axil cells that have lost *STM* expression can respond to ectopic STM activity. Firstly, we detected a dramatically increase of *STM* expression by reverse transcription quantitative PCR (RT-qPCR) in leaf-removed leaf axil-enriched shoot apex tissues (Fig 3T). In mature leaf axils, we found that, following Dex induction, no axillary bud could form (Fig 3U, 3V and 3Y), highlighting the importance of the low level *STM* expression for subsequent AM initiation. Similarly, when we introduced *p35S*::*STM-GR* into *stm-bum1* plants with compromised AM initiation, we found that Dex treatment did not induce axillary buds from mature leaf axils (Fig 3Y). Taken together, these results indicate that the recently identified leaf axil auxin minimum is required to maintain low level *STM* expression, which is then required for later axillary buds formation.

### AM Initiation Requires *REV*-dependent Up-Regulation of *STM* Expression

**Fig 4:**
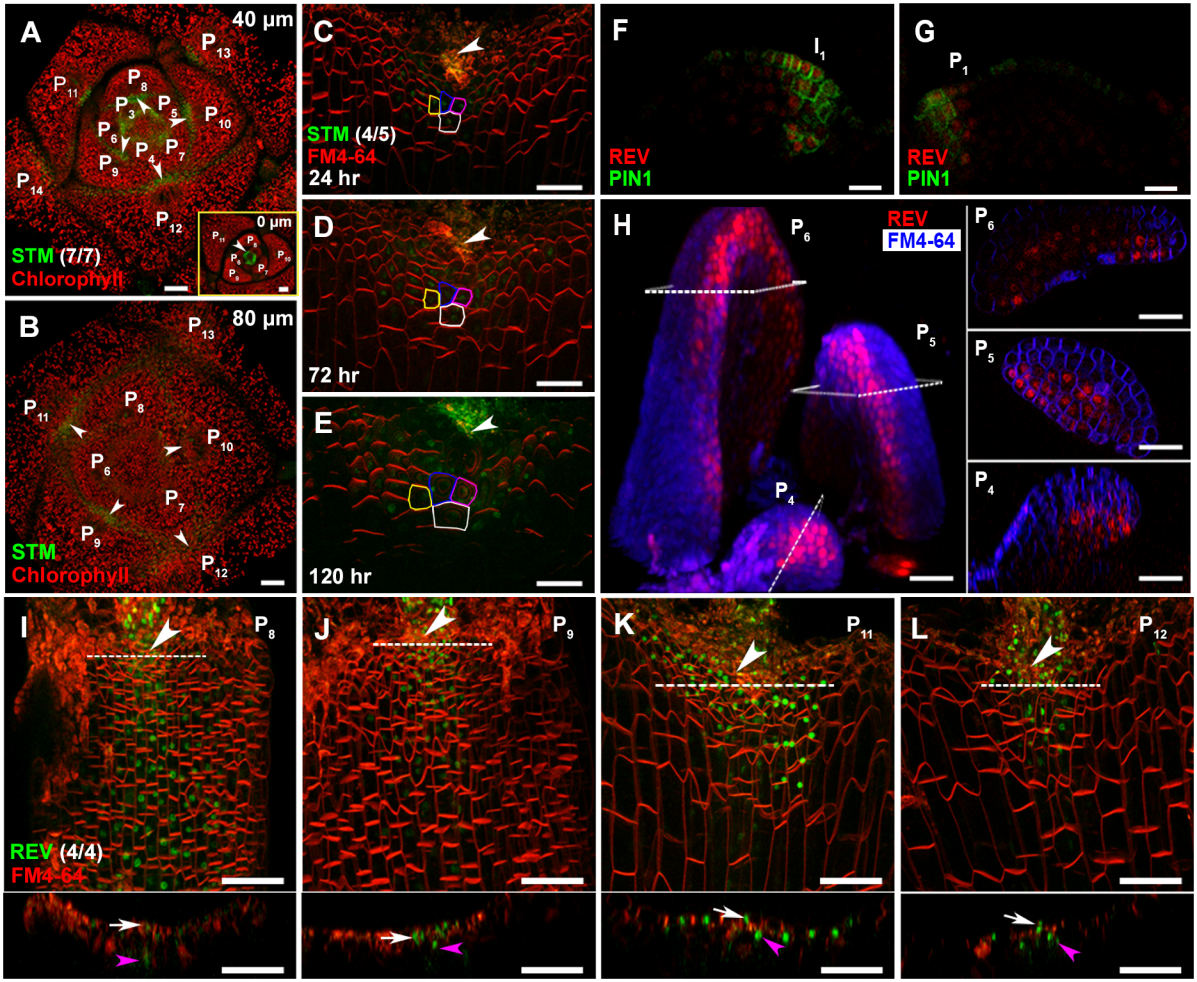
*STM* expression in *rev-6* and *REV* expression in the leaf axil. (A and B) Continuous transverse sections through meristem and leaf primordia regions showing *STM-Venus* (green) expression in *rev-6* leaf axils (arrowheads). (A) is more apical to (B) with the most apical section inserted at the bottom left-hand corner, and approximate distance (in micrometers) from the summit of the SAM to the section is given in the upper right-hand corner of each image. Note detectable STM-Venus signal in all leaf axils, which is reduced in P_10_ and older leaves. (C-E) Reconstructed view of the L1 layer of a *rev-6* leaf axil (as shown in Fig 1B) with STM-Venus (green) expression and FM4-64 stain (red) showing the location of the STM-positive cells, with (C) being the first time point and elapsed time in (D and E). Selected progenitor cells are color-coded, and the same color has been used for each progenitor cell and its descendants. Arrowheads highlight the approximate center of the incision line. (F-H) Spatial dynamics of REV distribution in early leaf primordia. (F and G) Continuous longitudinal sections through a vegetative shoot apex showing expression of REV-Venus (red) extends to the adaxial domain of I_1_ and P_1_. There is also low levels of expression of REV-Venus in the meristem. PIN1-GFP expression and localization are shown in green. (H) Optical transverse sections through a vegetative shoot apex showing expression of REV-Venus (red) in the adaxial domain of P_4_, P_5_ and P_6_. (I-L) Reconstructed view of L1 layer of a leaf axil with REV-Venus (green) expression and FM4-64 stain (red) showing REV distribution in P_8_ (I), P_9_ (J), P_11_ (K), and P_12_ (L) leaves. Optical longitudinal sections of the leaf axil region along the planes of sections, as depicted by dotted lines, are shown below the corresponding images. Note that *REV* is highly expressed in the leaf axil center in P_9_ and older leaves. White arrows indicate epidermal expression of REV-Venus, and pink arrowheads indicate subepidermal expression. Arrowheads highlight the approximate center of the incision line. Bars = 50 μm in (A-E and I-L) and 20 μm in (F-H).

In contrast to *pCUC2>>iaaM* and *pLAS*::*iaaM-en* plants, we found that *STM* expression was maintained in the *rev-6* mutant (Fig 4A–4E), which also lacks axillary buds [43]. In contrast to the wild type (Fig 1K–1M), the expression of *STM* does not increase in the *rev-6* mutant (Fig 4A and 4B), as it does in wild-type leaf axils, during leaf maturation (compare Figs 1B–1G and 4C–4E). Subsequently, the *STM*-expressing cells did not undergo active cell division to form a meristem with well-organized structure (Fig 4C–4E). The change of leaf axil *STM* expression in *rev-6* implies that up-regulation of *STM* expression in P_10_ and older leaves requires REV, but maintenance of *STM* expression does not require REV. Also in contrast to *stm-bum1* and *pCUC2>>iaaM*, our genetic analysis indicates that over-expressing *STM* can suppress the AM initiation defect of *rev-6* mutants (Fig 3W–3Y). Therefore, we conclude that *STM* expression must not only be maintained in meristematic cells, but also subsequently up-regulated for AM initiation.

To test if REV up-regulates *STM* expression in a cell-autonomous manner, we imaged REV distribution by using a functional *pREV*::*REV-Venus* reporter line [32]. REV-Venus is broadly expressed in the adaxial side of P_8_ and younger leaves (Fig 4F–4I), but it is restricted to the center of leaf axils, especially the epidermis (L1) layer, in P_9_ and older leaves (Fig 4J–4L). Furthermore, *REV* has stronger expression in P_9_ and older leaf axils than in younger leaf axils. The leaf axil enrichment of REV is consistent with up-regulated *STM* expression in P_10_ and older leaves, suggesting that REV up-regulates *STM* expression in a cell-autonomous manner.

Overexpressing alleles of *REV* and related HD-ZIPIII genes can induce ectopic AMs in the abaxial side leaf axils [14, 44, 45]. By using one such mutant, *phavulota-1d* (*phv-1d*), we observed ectopic *STM* expression in abaxial leaf axils prior to axillary bud initiation (S3B–S3E Fig). By using transgenic lines overexpressing microRNA-insensitive *REV* and *PHABULOSA* (*PHB*), another related HD-ZIPIII gene, we detected up-regulation of *STM* expression in leaf-removed shoot apex tissues, which are enriched with leaf axils (S3J Fig). Notably, we also detected ectopic auxin minima in abaxial leaf axils by using the auxin concentration sensor DII-Venus [46], whose strong abaxial axil signal indicates low auxin concentrations (S3F–S3I Fig). Taken together, REV and related HD-ZIPIII proteins can promote *STM* expression, which, together with auxin minima, promote ectopic AM initiation.

### REV Directly Up-Regulates *STM* Expression

**Fig 5:**
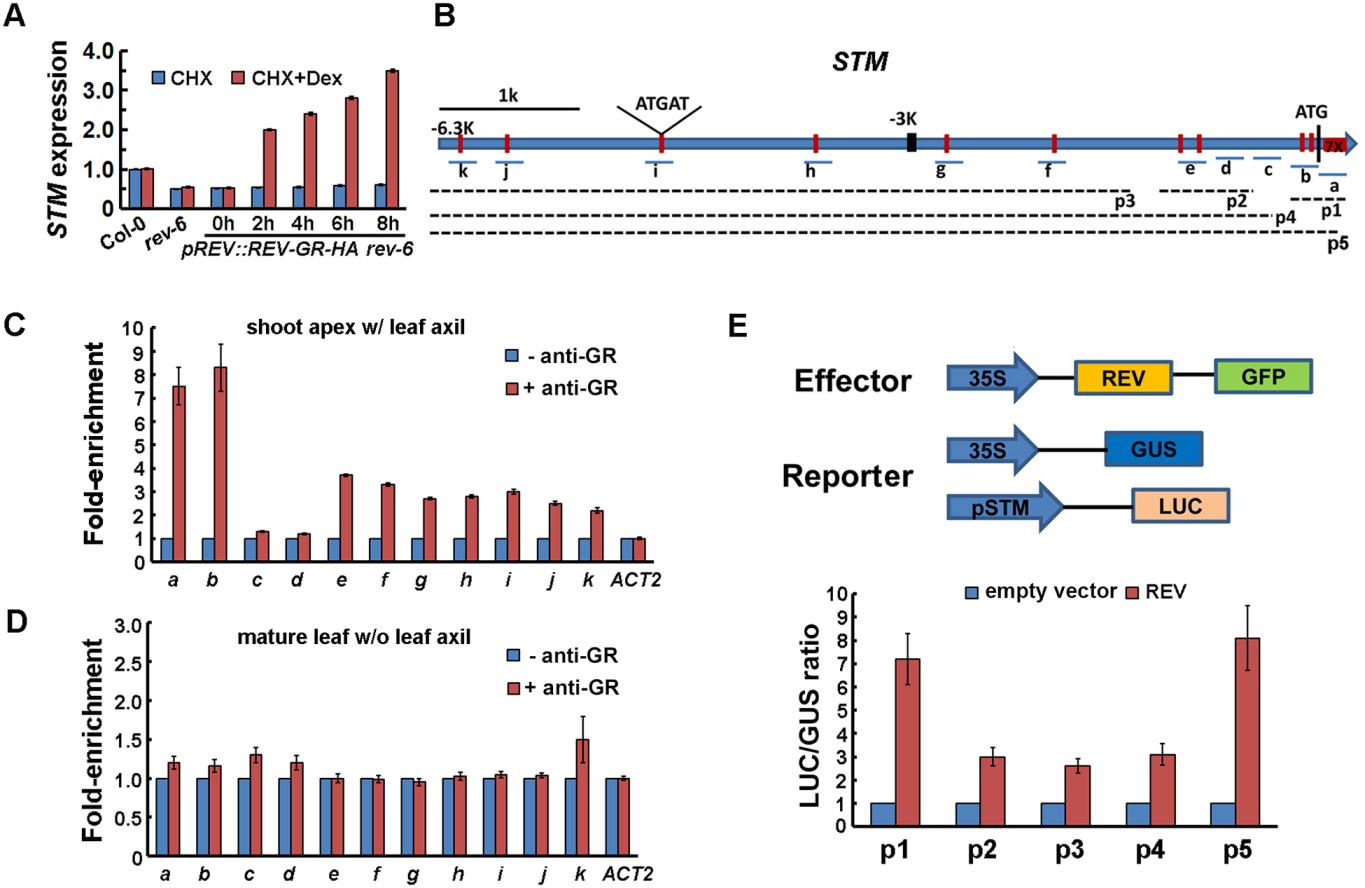
Direct up-regulation of *STM* expression by REV. (A) RT-qPCR analysis of *STM* expression in *pREV*::*REV-GR-HA rev-6* vegetative shoot apex tissues (with leaves removed) before and after simultaneous Dex and CHX treatment. The vertical axis indicates relative mRNA amount compared with the amount before treatment. Error bars indicate SD. (B) Schematic diagram of the *STM* genomic region. Vertical red lines indicate the sites containing the consensus REV binding sequence (ATGAT box). ATG denotes the translation start site. The underlying lines represent the DNA fragments amplified in ChIP assays, or used for plant protoplast assays. (C and D) ChIP enrichment test by PCR shows binding of REV-GR-HA to the ATGAT box-containing regions, especially the ones near the start site, in vegetative shoot apex tissues enriched with leaf axil (C) but not mature leaves (>P_10_) without the leaf axil region from 30-d old plants (D) of *pREV*::*REV-GR-HA rev-6* plants. A paired design was used, in which each measurement was paired with a corresponding control without antibody. Error bars indicate SD. More controls are shown in S4I Fig. (E) Transcriptional activity assays in *Arabidopsis* protoplasts. A *p35S*::*GFP* empty vector was the negative control, and a *p35S*::*GUS* line was the internal control. Relative *LUC* reporter gene expression is shown in the lower panel. The p1-p5 regions (indicated as in B) were assayed. Data are mean ± SD. Error bars are derived from three independent biological experiments, each run in triplicate.

To test if REV directly up-regulates *STM* expression, we generated functional Dex-inducible *pREV*::*REV-GR-HA rev-6* lines (S4A–S4C Fig). We measured the effect of REV activation on the expression of *STM* by RT-qPCR. REV activation resulted in rapid elevation of *STM* mRNA levels within 2 h of treatment, with or without the protein synthesis inhibitor cycloheximide (CHX) (Fig 5A and S4D Fig), strongly suggesting that induction of *STM* does not require *de novo* protein synthesis and that *STM* is likely a direct target of REV. REV activation also triggered *in vivo* accumulation of STM-Venus, as shown by live-cell imaging (S4E–S4H Fig). Consistent with this, our recent large-scale yeast one-hybrid assay identified REV and related HD-ZIPIII proteins as binding to the *STM* promoter region [21].

We next performed chromatin immunoprecipitation (ChIP) assays to examine whether REV directly binds to the *STM* promoter *in vivo*. We scanned the *STM* genomic sequence for ATGAT, the conserved binding site for REV [47], and designed primers near identified motifs and other regions (Fig 5B). In both shoot apex tissues enriched with leaf axils and inflorescence tissues, we found that REV-GR-HA strongly associated with the regions containing multiple ATGAT motifs, but only after Dex treatment, by using antibodies against GR or HA (Fig 5C and S4I Fig). In addition, REV-GR-HA weakly associated with seven other upstream ATGAT motif-containing regions. A transient transfection assay in protoplasts further confirmed that REV bound to multiple ATGAT motif-containing *STM* genomic regions, especially the ones close to the start codon, and up-regulated *STM* expression (Fig 5E). These newly transformed *pSTM*::*LUC* constructs would lack epigenetic modifications that might interfere with REV binding.

### Cell Type-Specific REV Binding to the *STM* Region

**Fig 6:**
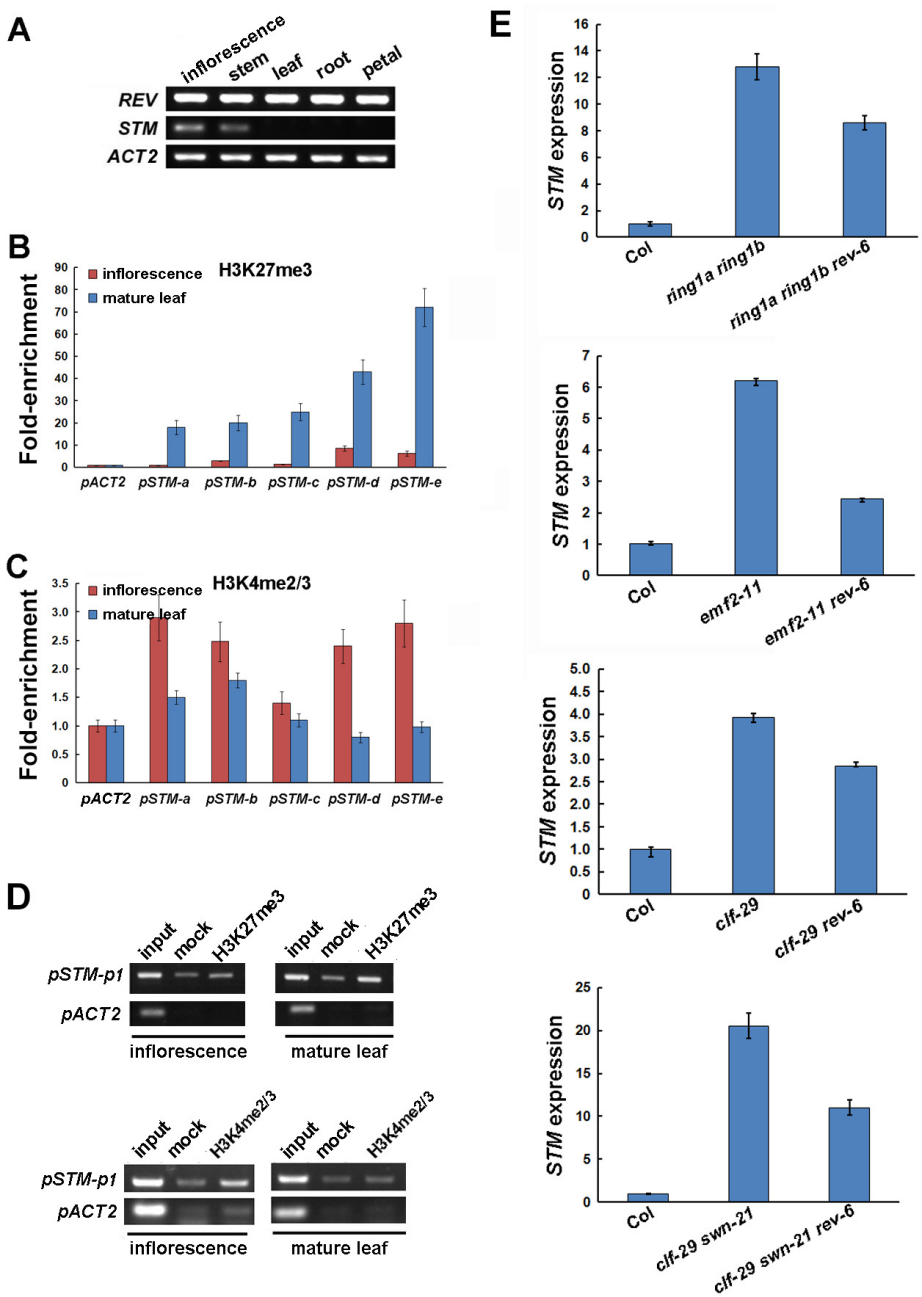
Epigenetic modification of the *STM* locus. (A) RT-PCR with primers amplifying the *REV*, *STM*, or *ACT2* coding regions on cDNAs generated from mRNA isolated from Col-0 wild-type inflorescence, stem, mature leaf (without the leaf axil region), root, and petal, respectively. *ACT2* was used as a loading control. (B and C) Results of ChIP-qPCR performed on IP with antibodies against H3K27me3 (B) and H3K4me2/3 (C) on chromatin samples extracted from Col-0 wild-type inflorescences and mature leaves. The a-e regions (indicated as in Fig 5B) were assayed. Error bars indicate SD. More controls are shown in (D). (D) ChIP enrichment test by PCR with an anti-H3K27me3 antibody and an anti-H3K4me2/3 antibody using Col-0 wild-type inflorescences and mature leaves, together with total DNA input (input) and no-antibody (mock) controls. An *ACT2* promoter region was used as a negative control. (E) Up-regulation of *STM* expression in mutants affecting PRC1 and PRC2 requires *REV*. RT-qPCR analysis of *STM* in whole seedlings of Col-0 wild type and mutants affecting PRC1 and PRC2 w/ or w/o *rev-6*. The vertical axis indicates relative mRNA amount compared with the amount in wild-type plants. Error bars indicate SD.

REV is widely expressed in young leaves, including in the adaxial domain and vascular tissues [43], but only up-regulates *STM* expression in boundary tissues-enriched samples (Fig 6A), as previously shown [11]. Furthermore, a recent ChIP-seq analysis did not identify *STM* as a REV-binding target in whole seedlings [47]. By using the same antibodies and protocol, we found that REV associated with the *STM* genomic region only in vegetative shoot apex and inflorescence tissues, but not in leaf axil region-removed mature leaves (compare Fig 5C and 5D, only data from vegetative shoot apex tissues was shown).

In animals, lineage-specific epigenetic modification of transcription factor genes leads to the fixation of stem cell fate [48]. Furthermore, the *STM* locus was epigenetically silenced in mature leaves containing only differentiated cells [49–51]. In mature leaves without *STM*-expressing leaf axil cells, the chromatin modification H3K27me3, which is associated with transcriptional repression, is highly enriched at the *STM* locus (Fig 6B and 6D). By contrast, H3K4me2 and/or H3K4me3, which are associated with transcriptional activation, are enriched at the *STM* locus in inflorescence tissues enriched with organ axils (Fig 6C and 6D). This histone modification pattern implies that epigenetic factors may regulate REV binding to the *STM* locus.

In both animals and plants, the Polycomb Repressive Complex 2 (PRC2) establishes the H3K27me3 mark, which provides a docking site for PRC1 to establish a repressive chromatin configuration [52]. Mutants affecting PRC1 and PRC2 have elevated *STM* expression [49, 50]. To test if the ectopic activation of *STM* expression requires REV, we introduced *rev-6* into PRC mutants. We found that *rev-6* mutation partly suppressed ectopic *STM* up-regulation (Fig 6E).

## Discussion

### A Meristematic Cell Population in the Leaf Axil

Plant cells, especially isolated cells, have amazing developmental plasticity, yet intact plant development follows defined patterning. Within meristems, clonal analysis and root regeneration studies suggest that meristem cells usually lack predictable destinies and that positional control is most important for plant cell fate determination [38–41]. However, distinct cell lineages emerge at later developmental stages [2, 3]. In this study, we show that AM initiation is accompanied by the maintenance of a meristematic cell population, and differentiation of surrounding cells. We traced this cell population in P_6_ and older leaves, and confirmed that *STM*-positive cells at the leaf axil are progenitors of axillary buds. Imaging results indicate that cells usually cannot acquire *STM* expression *de novo* (Fig 1B–1G), at least in P_6_ and older leaves, indicating the existence of a cell lineage. Further laser ablation results show that these *STM*-positive cells are necessary for formation of axillary buds, whereas neighboring *STM*-negative cells are differentiated (Fig 2B–2G).

This leaf axil meristematic cell population relies on positional cues. Our recent studies have shown that an auxin minimum, which is associated with the leaf axil position, is required for AM initiation [9, 27]. In the current work, we further demonstrate that the maintenance of the meristematic cells depends on low auxin (Fig 3G–3J and 3P–3S), which is likely determined by positional information. The observation of abaxial auxin minima and *STM* expression in *phv-1d*, which forms axillary buds at the abaxial side, also support the importance of positional cues for the maintenance of meristematic cells (S3B–S3I Fig).

Cell fate determination occurs gradually with cell cycle progression in animals [48]. Previous studies focused on cells within shoot meristems and root meristems, and found that cells from different meristematic domains can switch cell fate [38–41]. These results do not necessarily indicate that cell fate determination does not occur after additional rounds of cell cycle progression. In fact, root meristem regeneration does not occur if additional tissue beyond the meristematic zone has been removed [53]. If one assumes that cell fate determination takes place after more cell cycles in plants (than in animals), it would be conceivable that: i) cells within or close to meristems remain meristematic and can reverse cell fate, and ii) certain non-dividing or slow-dividing cell types in differentiated organs may maintain a meristematic status while their neighboring cells become fully differentiated and can no longer reverse to a meristematic status. Because boundary cells are non-dividing or slow-dividing cells [54], leaf axil cells can maintain an undifferentiated status while their neighboring differentiated cells cannot.

### Cell Fate Determination in Differentiated Cells

Previous studies have shown that overexpression of *STM* (or the related gene *KNAT1*) alone [55–57], or in combination with ectopic *WUS* [42, 58], induces ectopic meristems. The effect of ectopic STM is highly dependent on tissue stage. As shown in a previous works [57], leaf primordia older than P_10_ are not competent to ectopic STM activity (S5A Fig). For younger leaf primordia, ectopic meristems initiated only from leaf axils and the adaxial side of the proximal portion of leaf blades, especially in the sinus region between the blade and the petiole (55 out of 72 P_7_ to P_9_, i.e. 72%, S5B–S5G Fig). Thus, STM alone is not sufficient to induce meristems from most cells, but is sufficient in presumably undifferentiated cells.

Similarly, we found that ectopic STM activity was insufficient to rescue axillary bud formation defects in mature leaf axils of *pCUC2>>iaaM*, *pLAS*::*iaaM-en*, or *stm-bum1* plants, which have lost low level *STM* expression in leaf axil cells. By contrast, ectopic STM activity was sufficient in *rev-6* maintaining low level *STM* expressing cells (Fig 3W and 3X). Therefore, *STM* expression and proper cell fate are both required for AM initiation. In tomato, recent work showed that ectopic meristems may form at the base of leaflets, where *KNOX* genes express, and this requires the AM initiation pathway [59]. This finding again supports the model that cell competency is required for shoot meristem formation. Epigenetic regulation is involved in the maintenance of meristematic cell competency, and *STM* expression serves as a marker for cell competency.

### A Threshold Model for AM Initiation

Our data support the detached model for AM initiation, in which meristematic cells are detached from the SAM. When leaf primordia (P_1_) separate from the SAM, boundary cells keep *STM* expression as SAM cells (Fig 1H). From P_1_ to P_5_ stages, the number of *STM*-expressing cells continues to decrease (Fig 1H–1N). Although the exact clonal relationship of early *STM*-expressing cells remains unknown, our data suggest that many *STM*-expressing cells differentiate but some may maintain *STM* expression. In P_6_ and older leaf primordia, we used live-cell imaging to track the *STM*-expressing cell population (Fig 1B–1G). Notably, all cells in the enlarged *STM*-expressing domain are progeny of cells with previous *STM* expression. Our data also explain the ectopic axillary formation of *phv-1d* mutants. Ectopic axillary buds form in the abaxial side away from the SAM, providing key support to the *de novo* model [14]. PHV is highly similar to REV, and can bind to the *STM* promoter region in yeast. It is conceivable that ectopic *PHV* expression in *phv-1d* would result in ectopic STM expression (S3E Fig), resulting in ectopic meristematic cells in the abaxial leaf axil that initiate ectopic axillary buds.

**Fig 7:**
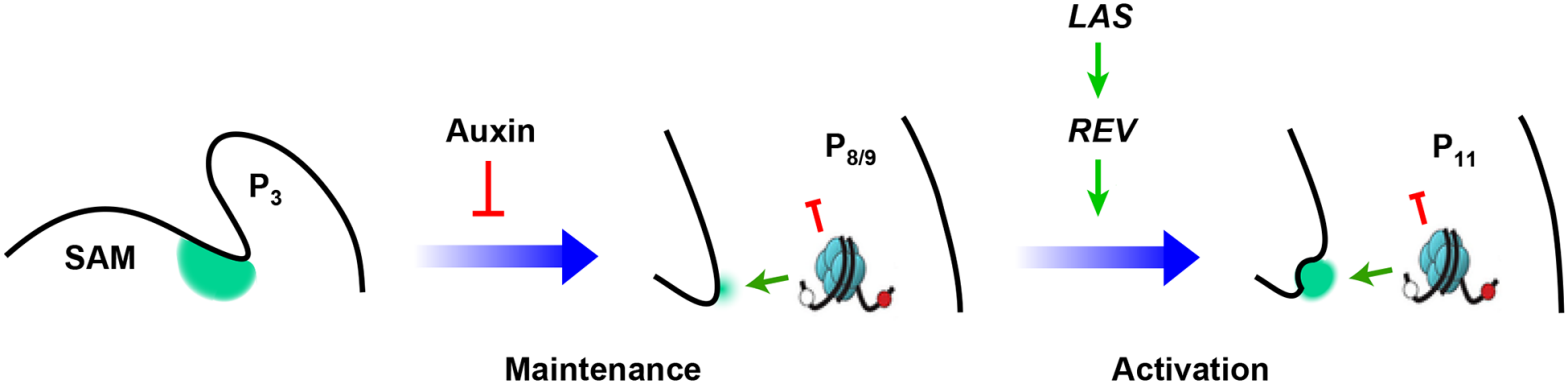
Conceptual model showing meristematic cells maintenance and up-regulation during AM initiation. Early leaf primordium axils maintain low levels of *STM* expression, which requires the leaf axil auxin minimum. In more mature leaf primordia, the expression of *REV*, which is under *LAS* regulation, up-regulate *STM* expression to promote AM initiation and subsequent axillary bud formation.

Furthermore, our results support a ‘threshold model’ in which maintenance of low levels of *STM* expression is required but not sufficient for AM initiation, and subsequent elevated expression of *STM* would induce AM initiation (Fig 7). Leaf axil cells show low auxin-dependent low levels of *STM* expression starting at leaf primordium initiation. The early low level *STM* expression is required for later AM formation (Fig 3). In addition, cells lost *STM* expression are no longer sensitive to ectopic STM activities at a later stage. Before AM initiation, *STM* is up-regulated in the center of the leaf axil, triggered by *REV* activation, which in turn requires *LAS* activity [11]. We also show that this up-regulation is a local event (Fig 4I–4L), and it depends on prior, maintained *STM* expression (S4E–S4H Fig). We further show that REV binding to the *STM* promoter is tissue-specific (Fig 5), and that epigenetic regulation may underlie this cell type specificity (Fig 6), suggesting that the binding requires permissive chromatin statues. Our data favor the idea that the up-regulation of *STM* is causal for AM initiation, rather than a consequence of a newly formed AM, because the expression of *WUS* and *CLV3* are still missing at the stage of initial *STM* up-regulation.

## Materials and Methods

### Plant Material, Generation of Transgenic Plants, and Pharmacological Treatment

The *Arabidopsis thaliana* ecotypes Landsberg *erecta* (L*er*) or Columbia (Col-0) were used as the wild type. The *atring1a atring1b*, *emf2-11*, *clf-29*, *clf-29 swn-21*, *rev-6*, *stm-bum1*, *p35S*::*REVm-MYC*, *p35S*::*PHBm-MYC* and *pREV*::*REV-GR-HA* lines are in the Col-0 background [37, 50]; the *pCLV3*::*GFP-ER pWUS*::*DsRed-N7*, *pREV*::*REV-Venus*, *pSTM*::*STM-Venus*, and *p35S*::*STM-GR* lines are in the L*er* background [32, 42, 60], and the J0121 line is in the Ws-0 background. In this study, we confirmed that the *pSTM*::*STM-Venus* reporter can rescue the *stm-11* mutant phenotype. Genotyping primers are listed in S1 Table. Plants were grown in the greenhouse on soil at 22°C under short-day conditions (8 h light/16 h dark) unless otherwise specified.

Leaf culture followed a previously described protocol [9]. Briefly, seedlings were grown in MS medium under short-day conditions for 15 d after seed stratification. Leaves between P_5_ and P_11_ were then detached from seedlings, laid flat on MS medium supplemented with 0.5 mg/L folic acid and 100 mg/L inositol, and grown for up to 30 d under the same conditions.

The *pREV*::*REV-GR-HA* construct was made by replacing the endogenous stop codon of TAC clone JAtY80N08 covering the *REV* genomic region with a GR-HA sequence using recombineering [61]. The construct was then transformed into *rev-6* plants. Over 20 transgenic lines were obtained and lines with stringently inducible rescue phenotypes were used.

### Confocal Microscopy, Optical Microscopy, and Scanning Electron Microscopy

Confocal microscopy images were taken with a Nikon A1 confocal microscope. Samples were either live-imaged or fixed and sectioned as previously described [9]. Excitation and detection wavelengths for GFP, Venus, and DsRed were as previously described [9, 32]. To detect FM4-64 and PI staining, a 514 nm laser line was used for excitation and a 561 nm long-pass filter was used for detection. The modified pseudo-Schiff-PI (mPS-PI) staining was performed as described and a 488 nm laser line was used for excitation and emission was collected at 520–720 nm [62]. DAPI staining was excited at 405 nm and detected in the 425–475 nm. Autofluorescence was excited at 488 nm or 514 nm and detected in the 660–700 nm range.

Optical photographs were taken with a Nikon SMZ1000 stereoscopic microscope or an Olympus BX60 microscope equipped with a Nikon DS-Ri1 camera. Scanning electron microscopy was performed using a Hitachi S-3000N variable pressure scanning electron microscope after standard tissue preparation [9].

### Laser Ablation

Laser ablations were performed on a Nikon A1 confocal microscope equipped with an Andor MicroPoint laser system consisting of a pulsed 440 nm nitrogen laser. We adjusted a variable neutral density filter to attenuate the output laser to limit damage to targeted cells, as assessed by confocal imaging. The observation of cell collapse was used to confirm successful ablation (Fig 2C and 2F).

### RT-PCR and RT-qPCR

Total RNA was extracted from leaves, shoot apex tissues, or inflorescences (~6 d after bolting) of 12 plants using the AxyPrep Multisource RNA Miniprep kit (Corning). For shoot apex tissues enriched for leaf axils, leaves were manually removed from 25 d plants grown under short day conditions. For induced meristems, total RNA was extracted from leaf sinus tissues using the RNAqueous-4PCR kit (Life Technologies). First-strand cDNA synthesis was performed with 2 μg total RNA using TransScript One-Step gDNA Removal and cDNA synthesis SuperMix (TransGen), or with 300 ng total RNA using SuperScript III reverse transcriptase (Life Technologies), and 22-mer oligo dT primers according to the manufacturer’s instructions. RT-PCR analysis was performed in a 20 μL reaction using Taq DNA polymerase (TianGen) and gene-specific primers (S1 Table). Reverse transcription quantitative PCR (RT-qPCR) was performed on a Bio-Rad CFX96 real-time PCR detection system with the KAPA SYBR FAST qPCR kit (KAPA Biosystems). Relative expression by RT-qPCR was normalized to *TUB6* (*At5g12250*). Gene-specific primers (S1 Table) were used to amplify and detect each gene. Error bars of RT-qPCR experiments in Figures are derived from three independent biological experiments, each run in triplicate.

### Chromatin Immunoprecipitation

Chromatin immunoprecipitation (ChIP) experiments were performed according to published protocols [28, 49]. Leaf axil-enriched shoot apex tissues (under short day conditions) or inflorescences (under long day conditions) of approximately 4-week-old Col-0 wild-type or *pREV*::*REV-GR-HA rev*-6 plants were used. Plant material (800 mg) was harvested and fixed with 1% (v/v) formaldehyde under vacuum for 10 min. Nuclei were isolated and lysed, and chromatin was sheared to an average size of 1000 bp by sonication. The sonicated chromatin served as input or positive control. Immunoprecipitations were performed with a polyclonal antibody against GR (Affinity Bioreagents, PA1-516), a monoclonal antibody against HA (Beyotime, AH158), a polyclonal antibody against H3K27me3 (Millipore, 07–449), or a polyclonal antibody against H3K4me2/3 (Abcam, ab8580). The precipitated DNA was isolated, purified, and used as a template for PCR. RT-PCR was performed as described above (S1 Table). The data are presented as degree of enrichment of *STM* genomic fragments. The amount of precipitated DNA used in each assay was determined empirically such that an equal amount of *ACT2* (*At3g18780*) was amplified. Two independent sets of biological samples were used.

### Protoplast Transient Expression Assay

To produce the effector constructs, full-length *REV* was amplified from *Arabidopsis* cDNA and inserted into the pBI221 vector to generate pBI221-AP1. To generate *STM* promoter-driven *LUC* reporter genes, *STM* promoter regions were amplified from *Arabidopsis* genomic DNA. PCR fragments were inserted into the corresponding sites of the YY96 vector to produce *pSTM*::*LUC* constructs (Fig 5E, and S1 Table for primers).

Isolation of *Arabidopsis* protoplasts and PEG-mediated transfection were performed as described previously [28]. The reporter construct, effector plasmid, and a *p35S*::*GUS* construct (internal control) were co-transformed into protoplasts. After transformation, the protoplasts were incubated at 23°C for 12–15 h. The protoplasts were pelleted and resuspended in 100 μL of 1 × CCLR buffer (Promega). For the GUS enzymatic assay, 5 μL of the extract was incubated with 50 μL of 4-methylumbelliferyl-β-d-glucuronide assay buffer (50 mM sodium phosphate pH 7.0, 1 mM β-d-glucuronide, 10 mM EDTA, 10 mM β-mercaptoethanol, 0.1% sarkosyl, 0.1% Triton X-100) at 37°C for 15 min, and the reaction was stopped by adding 945 μL of 0.2 M Na_2_CO_3_. For luciferase activity assays, 5 μL of the extract was mixed with 50 μL of luciferase assay substrate (Promega), and the activity was detected with a Modulus Luminometer/Fluometer with a luminescence kit. The reporter gene expression levels were expressed as relative LUC/GUS ratios. Error bars in Fig 5E are derived from three independent biological experiments, each run in triplicate.

## Supporting Information

S1 Fig Expression of potential meristematic cell markers in leaf axils. (A-C) An example showing how fluorescence intensity are measured. According to the 3D-version images, appropriate regions of STM expression at the leaf axil (red circles) are selected on sum projected images in which all the pixels are added. Note to avoid regions with positive STM-expression but not belonging to the leaf axils. Background intensity is determined by selecting a region (blue circles) next to the *STM*-expression region and multiplying the mean fluorescence of background readings by the area of STM-expression region. For each leaf axil, the corrected total cell fluorescence (CTCF) was then calculated by subtracting background fluorescence density from integrated density. To make data comparable between samples, relative value was transferred into from the above absolute value by setting value of P_9_ at 1. (D-F) Continuous transverse sections through a vegetative L*er* wild-type shoot apex showing expression of *pCLV3*::*GFP-ER* (green) and *pWUS*::*DsRed-N7* (red) in mature buds but not young leaf axils. Sections are ordered from most apical (D) to most basal (F); approximate distance (in micrometers) from the summit of the SAM to the section is given in the bottom left-hand corner of each image. White arrowheads indicate leaf axils with florescent protein signals. Note the earliest appearance of CLV3 and WUS signals in P_12_. (G) Longitudinal sections through J0121 leaf axils of vegetative SAMs showing lack of pericycle marker J0121 (green) in leaf axils. The white arrow indicates an axillary bud. Bars = 50 μm.
(TIF)

S2 Fig Axillary buds cannot initiate from differentiated cells in *in vitro* cultured leaves. (A) A rosette leaf of P_7_ from a Col-0 wild-type plant was isolated, sliced twice along the petiole, and cultured in MS media containing no exogenous hormone for 15 d or longer. Note axillary buds only initiated from the cross section containing the original leaf axil (C), and adventitious roots may initiate from the cross section closest to the blade (B). Bars = 1 mm.
(TIF)

S3 Fig *STM* expression and auxin minima are required for AM initiation. (A) A cartoon showing the imaging angle of the abaxial leaf axil; the red-boxed area corresponds to imaged regions in (C, E, G and I). The arrowhead highlights the abaxial leaf axil. (B-I) Detection of STM-Venus (C and E) and DII-Venus (G and I) expression in abaxial leaf axils of the first true leaf of sibling wild-type (C and G) and *phv-1d/+* (E and I) plants. Light microscopy images of the same plants are shown in B, D, F and H. The dotted lines mark the cotyledons edges and white arrowheads points to abaxial leaf axils. Note the ectopic STM-Venus and DII-Venus signals and smaller cell size in *phv-1d/+* abaxial leaf axils. (J) RT-qPCR analysis of *STM* expression level in leaf axil-enriched tissues of *p35S*::*REVm-MYC* and *p35*::*PHBm-MYC* transgenic plants. Error bars indicate SD. Bars = 1 mm in (B, D, F and H) and 50 μm in (C, E, G and I).
(TIF)

S4 Fig Inducible REV rescues AM initiation defects and STM up-regulation. (A-C) Rescue of the AM defect in *rev-6* by inducible REV activation. (A) Close-up of rosette leaf axils in Col-0 wild-type, *rev-6*, and *pREV*::*REV-GR-HA rev-6* after mock or Dex treatment. After germination, Dex was daily applied to all leaf axils. Note the presence or absence (arrows) of an axillary bud. (B) Schematic representation of axillary bud formation in leaf axils of Col-0 wild-type plants, *rev-6* plants, and *pREV*::*REV-GR-HA rev-6* plants after mock or Dex treatment. The thick black horizontal line represents the border between the youngest rosette leaf and the oldest cauline leaf. Each column represents a single plant and each square within a column represents an individual leaf axil. The bottom row represents the oldest rosette leaf axils, with progressively younger leaves above. Green indicates the presence of an axillary bud, yellow indicates the absence of an axillary bud, and red indicates the presence of a single leaf in place of an axillary bud in any particular leaf axil. (C) Nuclear accumulation of the REV-GR-HA fusion protein after mock or Dex treatments. Protein gel blot detection of the REV-GR-HA fusion protein using crude nuclear extracts isolated from Col-0 wild-type and *rev-6* plants, and *pREV*::*REV-GR-HA* plants after mock or Dex treatment. Samples were harvested 1 d after treatment. (D) RT-qPCR analysis of *STM* expression in *pREV*::*REV-GR-HA rev-6* vegetative shoot apex tissues enriched with leaf axils after mock and Dex treatment. The vertical axis indicates relative mRNA amount after Dex treatment compared with the amount after mock treatment. Error bars indicate SD. (E-H) *In vivo* activation of *STM* expression by REV in *pREV*::*REV-GR-HA rev-6* plants. Reconstructed view of the L1 layer of a leaf axil (as shown in Fig 1B) with STM-Venus (green) expression and FM4-64 stain (red) showing the location and lineage of AM progenitor cells, with (E) being the first time point before Dex induction and elapsed time in (F-H). Selected progenitor cells are color-coded, and the same color has been used for each progenitor cell and its descendants. Arrowheads in (E-H) highlight the cut edge. (I) Enrichment of *STM* promoter fragment (as indicated in Fig 5B) in Dex induced *pREV*::*REV-GR-HA rev-6* plants. ChIP was carried out with anti-HA or anti-GR antibody, together with total DNA input (input) and no-antibody (mock) controls. *STM* promoter fragment 1 (see Fig 5B) was analyzed using inflorescence tissues. An *APETALA1* (*AP1*) promoter region was used as a positive control for REV binding [47], and an *ACT2* promoter region was used as a negative control. Bars = 1 mm in (A) and 50 μm in (D-G).
(TIF)

S5 Fig STM activity is sufficient to induce meristem from selected meristematic cells but not differentiated cells. (A) Frequency of ectopic meristem initiation from leaf primordia of different stages. (B) Scanning electron microscopy of ectopic meristems at the sinus region between blade and petiole of a *p35S*::*STM-GR* leaf at stage P_9_ 19 d after Dex induction. Arrows highlight flattened leaves. (C) Scanning electron micrograph of a *p35S*::*STM-GR* rosette leaf at stage P_8_ 11 d after induction. Arrows indicate the bulged meristems. (D-G) Scanning electron microscopy of ectopic meristems of (D) a P_7_ 16 d after induction, (E) a P_9_ petiole 19 d after induction, and (F and G) an intact plant 19 d after induction. The image in (E) corresponds to the leaf petiole region in the box bordered by the white dotted line in the insert. (G) A magnified image of the region in the box bordered by the black dotted line in (F). Bars = 200 μm in (B-G).
(TIF)

S1 Table Primers used for genotyping and expression analysis. (DOCX)

## References

[1] Birnbaum KD, Sanchez Alvarado A. Slicing across kingdoms: regeneration in plants and animals. Cell. 2008;132(4):697–710. Epub 2008/02/26. 10.1016/j.cell.2008.01.040 18295584 PMC2692308

[2] Lau OS, Bergmann DC. Stomatal development: a plant's perspective on cell polarity, cell fate transitions and intercellular communication. Development. 2012;139(20):3683–92. Epub 2012/09/20. 22991435 10.1242/dev.080523 PMC3445305

[3] Sugimoto K, Jiao Y, Meyerowitz EM. *Arabidopsis* regeneration from multiple tissues occurs via a root development pathway. Dev Cell. 2010;18(3):463–71. Epub 2010/03/17. 10.1016/j.devcel.2010.02.004 20230752

[4] Graham LE, Cook ME, Busse JS. The origin of plants: body plan changes contributing to a major evolutionary radiation. Proc Natl Acad Sci U S A. 2000;97(9):4535–40. Epub 2000/04/26. 10781058 10.1073/pnas.97.9.4535 PMC34322

[5] Coudert Y, Palubicki W, Ljung K, Novak O, Leyser O, Harrison CJ. Three ancient hormonal cues co-ordinate shoot branching in a moss. Elife. 2015;4. Epub 2015/03/26. 10.7554/eLife.06808 PMC4391503 25806686

[6] Domagalska MA, Leyser O. Signal integration in the control of shoot branching. Nat Rev Mol Cell Biol. 2011;12(4):211–21. Epub 2011/03/24. 10.1038/nrm3088 21427763

[7] Hagemann W. Comparative morphology of acrogenous branch systems and phylogenetic considerations. II. Angiosperms. Acta Biotheoretica. 1990;38:207–42.

[8] Wang Q, Hasson A, Rossmann S, Theres K. *Divide et impera*: boundaries shape the plant body and initiate new meristems. New Phytol. 2015;209(2):485–98. Epub 2015/09/24. 10.1111/nph.13641 26391543

[9] Wang Y, Wang J, Shi B, Yu T, Qi J, Meyerowitz EM, et al The stem cell niche in leaf axils is established by auxin and cytokinin in *Arabidopsis*. Plant Cell. 2014;26(5):2055–67. Epub 2014/05/21. 24850849 10.1105/tpc.114.123083 PMC4079368

[10] Long J, Barton MK. Initiation of axillary and floral meristems in *Arabidopsis*. Dev Biol. 2000;218(2):341–53. Epub 2000/02/05. 10656774 10.1006/dbio.1999.9572

[11] Greb T, Clarenz O, Schafer E, Müller D, Herrero R, Schmitz G, et al Molecular analysis of the *LATERAL SUPPRESSOR* gene in *Arabidopsis* reveals a conserved control mechanism for axillary meristem formation. Genes Dev. 2003;17(9):1175–87. Epub 2003/05/06. 12730136 10.1101/gad.260703 PMC196050

[12] Steeves TA, Sussex IM. Patterns in Plant Development. 2 ed Cambridge, UK: Cambridge University Press; 1989.

[13] Garrison R. Studies in the development of axillary buds. Am J Bot. 1955;42(3):257–66.

[14] McConnell JR, Barton MK. Leaf polarity and meristem formation in *Arabidopsis*. Development. 1998;125(15):2935–42. Epub 1998/07/10. 9655815 10.1242/dev.125.15.2935

[15] McSteen P, Hake S. *barren inflorescence2* regulates axillary meristem development in the maize inflorescence. Development. 2001;128(15):2881–91. Epub 2001/09/05. 11532912 10.1242/dev.128.15.2881

[16] Grbic V, Bleecker AB. Axillary meristem development in *Arabidopsis thaliana*. Plant J. 2000;21(2):215–23. Epub 2000/04/01. 10743661 10.1046/j.1365-313x.2000.00670.x

[17] Müller D, Schmitz G, Theres K. *Blind* homologous R2R3 Myb genes control the pattern of lateral meristem initiation in *Arabidopsis*. Plant Cell. 2006;18(3):586–97. Epub 2006/02/08. 16461581 10.1105/tpc.105.038745 PMC1383635

[18] Raman S, Greb T, Peaucelle A, Blein T, Laufs P, Theres K. Interplay of *miR164*, *CUP-SHAPED COTYLEDON* genes and *LATERAL SUPPRESSOR* controls axillary meristem formation in *Arabidopsis thaliana*. Plant J. 2008;55(1):65–76. Epub 2008/03/19. 10.1111/j.1365-313X.2008.03483.x 18346190

[19] Yang F, Wang Q, Schmitz G, Müller D, Theres K. The bHLH protein *ROX* acts in concert with *RAX1* and *LAS* to modulate axillary meristem formation in *Arabidopsis*. Plant J. 2012;71(1):61–70. Epub 2012/03/01. 10.1111/j.1365-313X.2012.04970.x 22372440

[20] Hibara K, Karim MR, Takada S, Taoka K, Furutani M, Aida M, et al *Arabidopsis CUP-SHAPED COTYLEDON3* regulates postembryonic shoot meristem and organ boundary formation. Plant Cell. 2006;18(11):2946–57. Epub 2006/11/24. 17122068 10.1105/tpc.106.045716 PMC1693926

[21] Tian C, Zhang X, He J, Yu H, Wang Y, Shi B, et al An organ boundary-enriched gene regulatory network uncovers regulatory hierarchies underlying axillary meristem initiation. Mol Syst Biol. 2014;10:755 Epub 2014/11/02. 10.15252/msb.20145470 25358340 PMC4299377

[22] Schumacher K, Schmitt T, Rossberg M, Schmitz G, Theres K. The *Lateral suppressor* (*Ls*) gene of tomato encodes a new member of the VHIID protein family. Proc Natl Acad Sci U S A. 1999;96(1):290–5. Epub 1999/01/06. 9874811 10.1073/pnas.96.1.290 PMC15132

[23] Schmitz G, Tillmann E, Carriero F, Fiore C, Cellini F, Theres K. The tomato Blind gene encodes a MYB transcription factor that controls the formation of lateral meristems. Proc Natl Acad Sci U S A. 2002;99(2):1064–9. Epub 2002/01/24. 11805344 10.1073/pnas.022516199 PMC117430

[24] Komatsu K, Maekawa M, Ujiie S, Satake Y, Furutani I, Okamoto H, et al *LAX* and *SPA*: major regulators of shoot branching in rice. Proc Natl Acad Sci U S A. 2003;100(20):11765–70. Epub 2003/09/18. 13130077 10.1073/pnas.1932414100 PMC208832

[25] Gallavotti A, Zhao Q, Kyozuka J, Meeley RB, Ritter MK, Doebley JF, et al The role of barren stalk1 in the architecture of maize. Nature. 2004;432(7017):630–5. Epub 2004/12/04. 15577912 10.1038/nature03148

[26] Li X, Qian Q, Fu Z, Wang Y, Xiong G, Zeng D, et al Control of tillering in rice. Nature. 2003;422(6932):618–21. Epub 2003/04/11. 12687001 10.1038/nature01518

[27] Wang Q, Kohlen W, Rossmann S, Vernoux T, Theres K. Auxin depletion from the leaf axil conditions competence for axillary meristem formation in *Arabidopsis* and tomato. Plant Cell. 2014;26(5):2068–79. Epub 2014/05/21. 24850851 10.1105/tpc.114.123059 PMC4079369

[28] Han Y, Zhang C, Yang H, Jiao Y. Cytokinin pathway mediates *APETALA1* function in the establishment of determinate floral meristems in *Arabidopsis*. Proc Natl Acad Sci U S A. 2014;111(18):6840–5. Epub 2014/04/23. 10.1073/pnas.1318532111 24753595 PMC4020066

[29] Liu J, Sheng L, Xu Y, Li J, Yang Z, Huang H, et al *WOX11* and *12* are involved in the first-step cell fate transition during *de novo* root organogenesis in *Arabidopsis*. Plant Cell. 2014;26(3):1081–93. Epub 2014/03/20. 10.1105/tpc.114.122887 24642937 PMC4001370

[30] Landrein B, Kiss A, Sassi M, Chauvet A, Das P, Cortizo M, et al Mechanical stress contributes to the expression of the *STM* homeobox gene in *Arabidopsis* shoot meristems. Elife. 2015;4:e07811 10.7554/eLife.07811 26623515 PMC4666715

[31] Kim JY, Yuan Z, Jackson D. Developmental regulation and significance of KNOX protein trafficking in *Arabidopsis*. Development. 2003;130(18):4351–62. Epub 2003/08/06. 12900451 10.1242/dev.00618

[32] Heisler MG, Ohno C, Das P, Sieber P, Reddy GV, Long JA, et al Patterns of auxin transport and gene expression during primordium development revealed by live imaging of the *Arabidopsis* inflorescence meristem. Curr Biol. 2005;15(21):1899–911. Epub 2005/11/08. 16271866 10.1016/j.cub.2005.09.052

[33] Reddy GV, Heisler MG, Ehrhardt DW, Meyerowitz EM. Real-time lineage analysis reveals oriented cell divisions associated with morphogenesis at the shoot apex of *Arabidopsis thaliana*. Development. 2004;131(17):4225–37. Epub 2004/07/29. 15280208 10.1242/dev.01261

[34] Clark SE, Jacobsen SE, Levin JZ, Meyerowitz EM. The *CLAVATA* and *SHOOT MERISTEMLESS* loci competitively regulate meristem activity in *Arabidopsis*. Development. 1996;122(5):1567–75. Epub 1996/05/01. 8625843 10.1242/dev.122.5.1567

[35] Endrizzi K, Moussian B, Haecker A, Levin JZ, Laux T. The *SHOOT MERISTEMLESS* gene is required for maintenance of undifferentiated cells in *Arabidopsis* shoot and floral meristems and acts at a different regulatory level than the meristem genes *WUSCHEL* and *ZWILLE*. Plant J. 1996;10(6):967–79. Epub 1996/12/01. 9011081 10.1046/j.1365-313x.1996.10060967.x

[36] Barton MK, Poethig RS. Formation of the shoot apical meristem in *Arabidopsis thaliana*: an analysis of development in the wild-type and in the *shoot meristemless* mutant. Development. 1993;119(3):823–31.

[37] Jasinski S, Piazza P, Craft J, Hay A, Woolley L, Rieu I, et al KNOX action in *Arabidopsis* is mediated by coordinate regulation of cytokinin and gibberellin activities. Curr Biol. 2005;15(17):1560–5. Epub 2005/09/06. 16139211 10.1016/j.cub.2005.07.023

[38] van den Berg C, Willemsen V, Hage W, Weisbeek P, Scheres B. Cell fate in the *Arabidopsis* root meristem determined by directional signalling. Nature. 1995;378(6552):62–5. Epub 1995/11/02. 7477287 10.1038/378062a0

[39] McDaniel CN, Poethig RS. Cell-lineage patterns in the shoot apical meristem of the germinating maize embryo. Planta. 1988;175(1):13–22. Epub 1988/07/01. 10.1007/BF00402877 24221624

[40] Jegla DE, Sussex IM. Cell lineage patterns in the shoot meristem of the sunflower embryo in the dry seed. Dev Biol. 1989;131(1):215–25. Epub 1989/01/01. 2909405 10.1016/s0012-1606(89)80053-3

[41] Reinhardt D, Frenz M, Mandel T, Kuhlemeier C. Microsurgical and laser ablation analysis of interactions between the zones and layers of the tomato shoot apical meristem. Development. 2003;130(17):4073–83. Epub 2003/07/23. 12874128 10.1242/dev.00596

[42] Gallois JL, Woodward C, Reddy GV, Sablowski R. Combined *SHOOT MERISTEMLESS* and *WUSCHEL* trigger ectopic organogenesis in *Arabidopsis*. Development. 2002;129(13):3207–17. Epub 2002/06/19. 12070095 10.1242/dev.129.13.3207

[43] Otsuga D, DeGuzman B, Prigge MJ, Drews GN, Clark SE. *REVOLUTA* regulates meristem initiation at lateral positions. Plant J. 2001;25(2):223–36. Epub 2001/02/13. 11169198 10.1046/j.1365-313x.2001.00959.x

[44] McConnell JR, Emery J, Eshed Y, Bao N, Bowman J, Barton MK. Role of *PHABULOSA* and *PHAVOLUTA* in determining radial patterning in shoots. Nature. 2001;411(6838):709–13. Epub 2001/06/08. 11395776 10.1038/35079635

[45] Emery JF, Floyd SK, Alvarez J, Eshed Y, Hawker NP, Izhaki A, et al Radial patterning of *Arabidopsis* shoots by class III HD-ZIP and KANADI genes. Curr Biol. 2003;13(20):1768–74. Epub 2003/10/17. 14561401 10.1016/j.cub.2003.09.035

[46] Vernoux T, Brunoud G, Farcot E, Morin V, Van den Daele H, Legrand J, et al The auxin signalling network translates dynamic input into robust patterning at the shoot apex. Mol Syst Biol. 2011;7:508 Epub 2011/07/08. 10.1038/msb.2011.39 21734647 PMC3167386

[47] Brandt R, Salla-Martret M, Bou-Torrent J, Musielak T, Stahl M, Lanz C, et al Genome-wide binding-site analysis of REVOLUTA reveals a link between leaf patterning and light-mediated growth responses. Plant J. 2012;72:31–42. Epub 2012/05/15. 10.1111/j.1365-313X.2012.05049.x 22578006

[48] Hemberger M, Dean W, Reik W. Epigenetic dynamics of stem cells and cell lineage commitment: digging Waddington's canal. Nat Rev Mol Cell Biol. 2009;10(8):526–37. Epub 2009/07/16. 10.1038/nrm2727 19603040

[49] Schubert D, Primavesi L, Bishopp A, Roberts G, Doonan J, Jenuwein T, et al Silencing by plant Polycomb-group genes requires dispersed trimethylation of histone H3 at lysine 27. EMBO J. 2006;25(19):4638–49. Epub 2006/09/08. 16957776 10.1038/sj.emboj.7601311 PMC1590001

[50] Xu L, Shen WH. Polycomb silencing of *KNOX* genes confines shoot stem cell niches in *Arabidopsis*. Curr Biol. 2008;18(24):1966–71. Epub 2008/12/23. 10.1016/j.cub.2008.11.019 19097900

[51] Katz A, Oliva M, Mosquna A, Hakim O, Ohad N. FIE and CURLY LEAF polycomb proteins interact in the regulation of homeobox gene expression during sporophyte development. Plant J. 2004;37(5):707–19. Epub 2004/02/12. 14871310 10.1111/j.1365-313x.2003.01996.x

[52] Zheng B, Chen X. Dynamics of histone H3 lysine 27 trimethylation in plant development. Curr Opin Plant Biol. 2011;14(2):123–9. Epub 2011/02/19. 10.1016/j.pbi.2011.01.001 21330185 PMC3081887

[53] Feldman LJ. The *de novo* origin of the quiescent center regenerating root apices of *Zea mays*. Planta. 1976;128(3):207–12. Epub 1976/01/01. 10.1007/BF00393230 24430748

[54] Breuil-Broyer S, Morel P, de Almeida-Engler J, Coustham V, Negrutiu I, Trehin C. High-resolution boundary analysis during *Arabidopsis thaliana* flower development. Plant J. 2004;38(1):182–92. Epub 2004/04/01. 15053771 10.1111/j.1365-313X.2004.02026.x

[55] Scofield S, Dewitte W, Nieuwland J, Murray JA. The *Arabidopsis* homeobox gene *SHOOT MERISTEMLESS* has cellular and meristem-organisational roles with differential requirements for cytokinin and CYCD3 activity. Plant J. 2013;75(1):53–66. Epub 2013/04/12. 10.1111/tpj.12198 23573875

[56] Chuck G, Lincoln C, Hake S. *KNAT1* induces lobed leaves with ectopic meristems when overexpressed in *Arabidopsis*. Plant Cell. 1996;8(8):1277–89. Epub 1996/08/01. 8776897 10.1105/tpc.8.8.1277 PMC161241

[57] Brand U, Grunewald M, Hobe M, Simon R. Regulation of *CLV3* expression by two homeobox genes in *Arabidopsis*. Plant Physiol. 2002;129(2):565–75. Epub 2002/06/18. 12068101 10.1104/pp.001867 PMC161677

[58] Lenhard M, Jurgens G, Laux T. The *WUSCHEL* and *SHOOTMERISTEMLESS* genes fulfil complementary roles in *Arabidopsis* shoot meristem regulation. Development. 2002;129(13):3195–206. Epub 2002/06/19. 12070094 10.1242/dev.129.13.3195

[59] Rossmann S, Kohlen W, Hasson A, Theres K. *Lateral suppressor* and *Goblet* act in hierarchical order to regulate ectopic meristem formation at the base of tomato leaflets. Plant J. 2015;81(6):837–48. Epub 2015/02/03. 10.1111/tpj.12782 25641652

[60] Li S, Liu L, Zhuang X, Yu Y, Liu X, Cui X, et al MicroRNAs inhibit the translation of target mRNAs on the endoplasmic reticulum in *Arabidopsis*. Cell. 2013;153(3):562–74. 10.1016/j.cell.2013.04.005 23622241 PMC3694718

[61] Zhou R, Benavente LM, Stepanova AN, Alonso JM. A recombineering-based gene tagging system for *Arabidopsis*. Plant J. 2011;66(4):712–23. Epub 2011/02/08. 10.1111/j.1365-313X.2011.04524.x 21294796

[62] Serrano-Mislata A, Schiessl K, Sablowski R. Active control of cell size generates spatial detail during plant organogenesis. Curr Biol. 2015;25(22):2991–6. 10.1016/j.cub.2015.10.008 26526374 PMC4651904

