# Supplementary figures and images for: Two-Step Regulation of a Meristematic Cell Population Acting in Shoot Branching in Arabidopsis
Source: PLoS Genet. 2016 Jul 11;12(7):e1006168. doi: 10.1371/journal.pgen.1006168 (PMC4939941; doi:10.1371/journal.pgen.1006168)

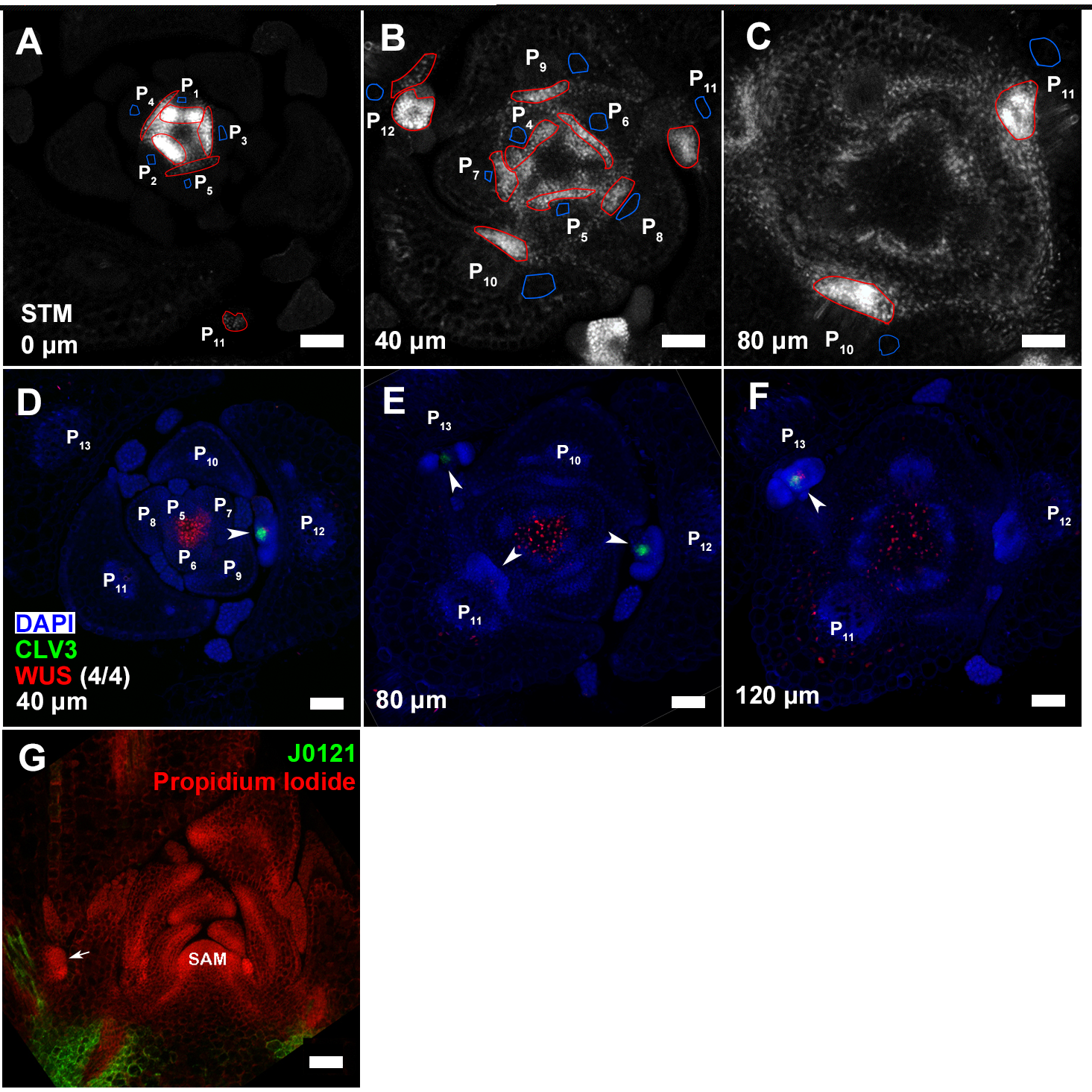

Supplement: S1 Fig — (A-C) An example showing how fluorescence intensity are measured. According to the 3D-version images, appropriate regions of STM expression at the leaf axil (red circles) are selected on sum projected images in which all the pixels are added. Note to avoid regions with positive STM-expression but not belonging to the leaf axils. Background intensity is determined by selecting a region (blue circles) next to the STM-expression region and multiplying the mean fluorescence of background readings by the area of STM-expression region. For each leaf axil, the corrected total cell fluorescence (CTCF) was then calculated by subtracting background fluorescence density from integrated density. To make data comparable between samples, relative value was transferred into from the above absolute value by setting value of P9 at 1. (D-F) Continuous transverse sections through a vegetative Ler wild-type shoot apex showing expression of pCLV3::GFP-ER (green) and pWUS::DsRed-N7 (red) in mature buds but not young leaf axils. Sections are ordered from most apical (D) to most basal (F); approximate distance (in micrometers) from the summit of the SAM to the section is given in the bottom left-hand corner of each image. White arrowheads indicate leaf axils with florescent protein signals. Note the earliest appearance of CLV3 and WUS signals in P12. (G) Longitudinal sections through J0121 leaf axils of vegetative SAMs showing lack of pericycle marker J0121 (green) in leaf axils. The white arrow indicates an axillary bud. Bars = 50 μm. (TIF) [file pgen.1006168.s001.tif]

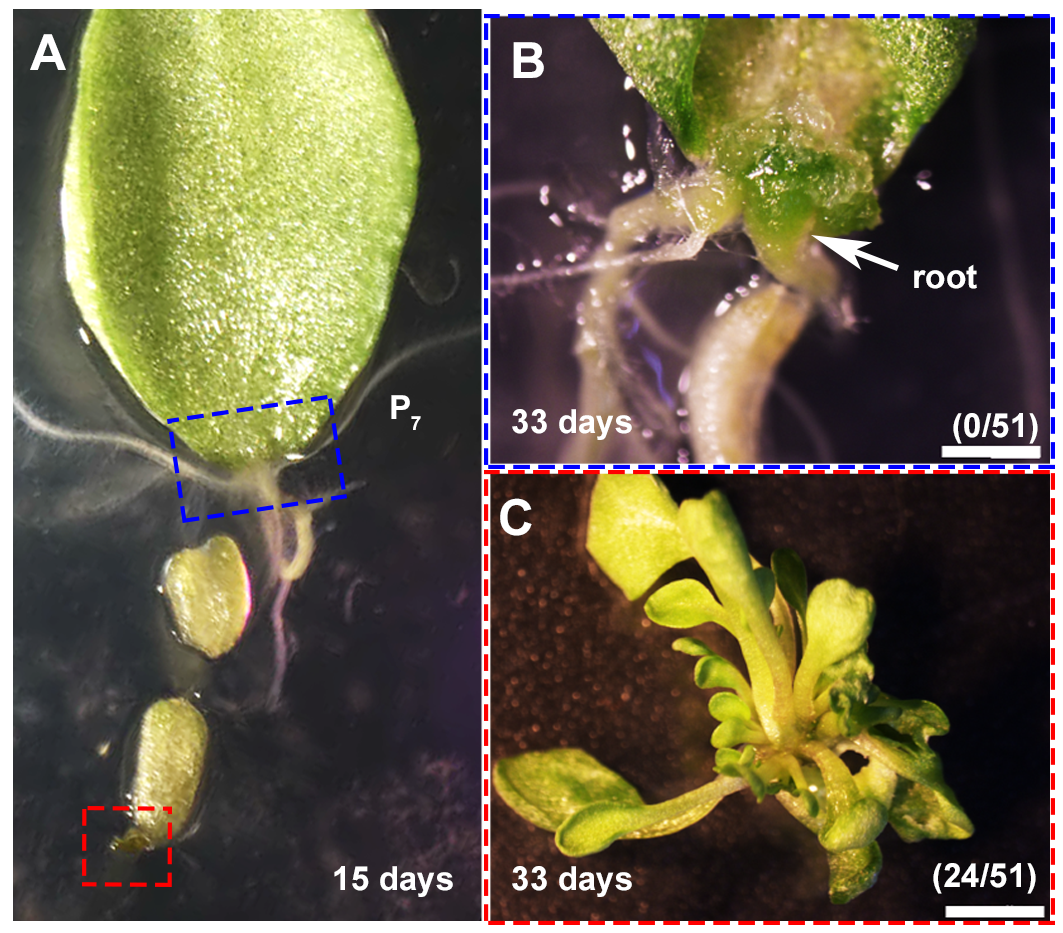

Supplement: S2 Fig — (A) A rosette leaf of P7 from a Col-0 wild-type plant was isolated, sliced twice along the petiole, and cultured in MS media containing no exogenous hormone for 15 d or longer. Note axillary buds only initiated from the cross section containing the original leaf axil (C), and adventitious roots may initiate from the cross section closest to the blade (B). Bars = 1 mm. (TIF) [file pgen.1006168.s002.tif]

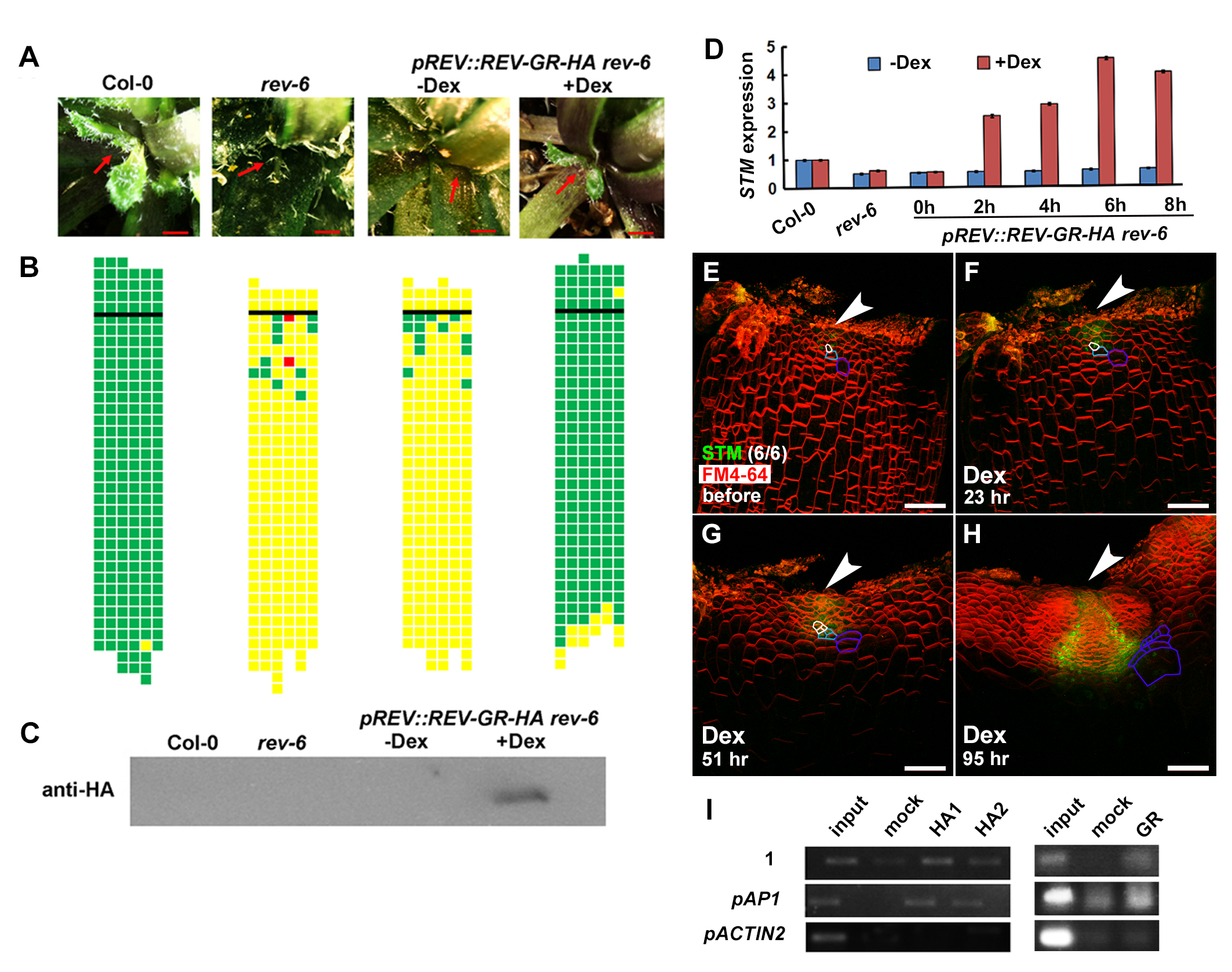

Supplement: S4 Fig — (A-C) Rescue of the AM defect in rev-6 by inducible REV activation. (A) Close-up of rosette leaf axils in Col-0 wild-type, rev-6, and pREV::REV-GR-HA rev-6 after mock or Dex treatment. After germination, Dex was daily applied to all leaf axils. Note the presence or absence (arrows) of an axillary bud. (B) Schematic representation of axillary bud formation in leaf axils of Col-0 wild-type plants, rev-6 plants, and pREV::REV-GR-HA rev-6 plants after mock or Dex treatment. The thick black horizontal line represents the border between the youngest rosette leaf and the oldest cauline leaf. Each column represents a single plant and each square within a column represents an individual leaf axil. The bottom row represents the oldest rosette leaf axils, with progressively younger leaves above. Green indicates the presence of an axillary bud, yellow indicates the absence of an axillary bud, and red indicates the presence of a single leaf in place of an axillary bud in any particular leaf axil. (C) Nuclear accumulation of the REV-GR-HA fusion protein after mock or Dex treatments. Protein gel blot detection of the REV-GR-HA fusion protein using crude nuclear extracts isolated from Col-0 wild-type and rev-6 plants, and pREV::REV-GR-HA plants after mock or Dex treatment. Samples were harvested 1 d after treatment. (D) RT-qPCR analysis of STM expression in pREV::REV-GR-HA rev-6 vegetative shoot apex tissues enriched with leaf axils after mock and Dex treatment. The vertical axis indicates relative mRNA amount after Dex treatment compared with the amount after mock treatment. Error bars indicate SD. (E-H) In vivo activation of STM expression by REV in pREV::REV-GR-HA rev-6 plants. Reconstructed view of the L1 layer of a leaf axil (as shown in Fig 1B) with STM-Venus (green) expression and FM4-64 stain (red) showing the location and lineage of AM progenitor cells, with (E) being the first time point before Dex induction and elapsed time in (F-H). Selected progenitor cells are color-c [file pgen.1006168.s004.tif]

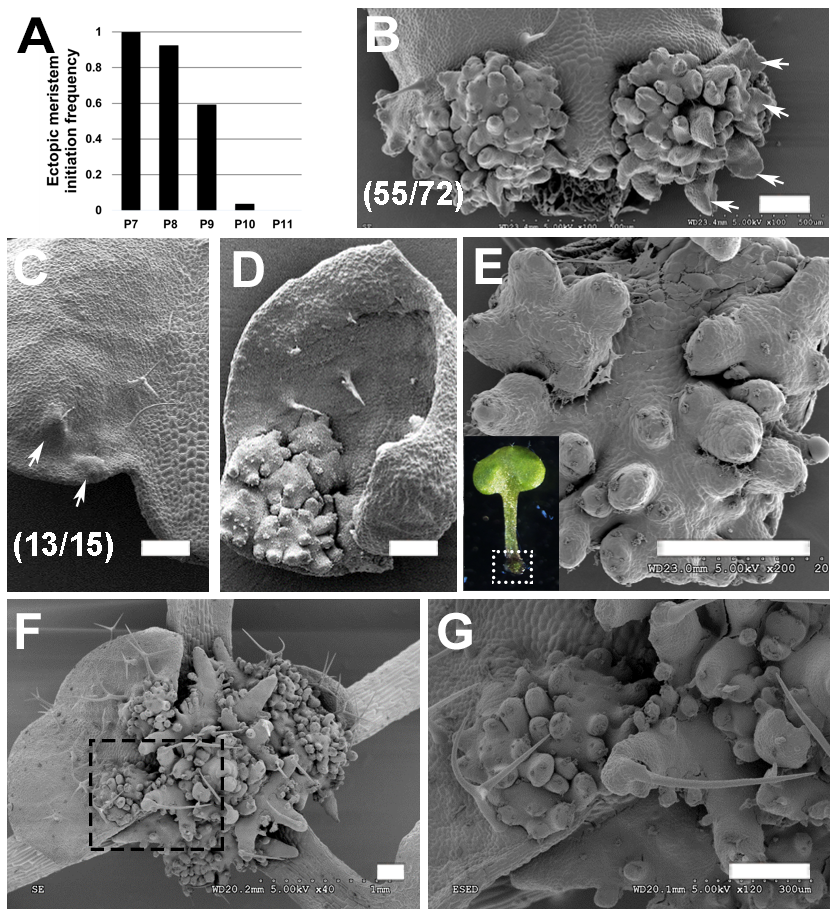

Supplement: S5 Fig — (A) Frequency of ectopic meristem initiation from leaf primordia of different stages. (B) Scanning electron microscopy of ectopic meristems at the sinus region between blade and petiole of a p35S::STM-GR leaf at stage P9 19 d after Dex induction. Arrows highlight flattened leaves. (C) Scanning electron micrograph of a p35S::STM-GR rosette leaf at stage P8 11 d after induction. Arrows indicate the bulged meristems. (D-G) Scanning electron microscopy of ectopic meristems of (D) a P7 16 d after induction, (E) a P9 petiole 19 d after induction, and (F and G) an intact plant 19 d after induction. The image in (E) corresponds to the leaf petiole region in the box bordered by the white dotted line in the insert. (G) A magnified image of the region in the box bordered by the black dotted line in (F). Bars = 200 μm in (B-G). (TIF) [file pgen.1006168.s005.tif]
