# Supplementary material for: Two-Step Regulation of a Meristematic Cell Population Acting in Shoot Branching in Arabidopsis
Source: PLoS Genet. 2016 Jul 11;12(7):e1006168. doi: 10.1371/journal.pgen.1006168 (PMC4939941; doi:10.1371/journal.pgen.1006168)
Supplement: S1 Table — (DOCX) [file pgen.1006168.s006.docx]

**S1 Table. Primers used for genotyping and expression analysis**

| Name | Sequence (5’-3’) |
| --- | --- |
| ring1a_FP2 | TAGTGAAACCCAACGACGCCG |
| ring1a_RP2 | GCTTCCTGCAAGCAGGACATTCG |
| ring1b-FP2 | TGACCAACTGGGTCGGAACTCAG |
| ring1b-RP2 | TCGAGCATTGCACGTCCTTACAG |
| emf2-11_FP2 | TCACTGTATGCGTCGTGGCAGAAA |
| emf2-11_RP2 | GAGGAGATCGTGGGTTGATGGC |
| clf-29_FP2 | TTCTTCGGCCACCAGATCGGAG |
| clf-29_RP2 | ACTGCAGATAAGCGCTTCCCCG |
| SWN-21-FP | CTGCTGCATCGAGCCGAGCTAC |
| SWN-21-RP | CCTCACCCATGCCATACTCCTGC |
| STM-FP2 | GGATCACATCTCCTCCACCATGC |
| STM-3UTR | GCTTGTTCATCAATCGGAACATG |
| GR-F | GAGGTGATTGAACCCGAGG |
| GR-R | ACCGTTGCCAGTTCTGAC |
| REV-F | GGGGTACCATGGAGATGGCGGTGGCT |
| REV-R | GTCTGCAGTCACACAAAAGACCAGTT |
| STM-qPCR-F | CTCCGGTTATGGAGAGACAGCAATTG |
| STM-qPCR-R | TCACCTTCCTCTTTCTCCGGTTATGG |
| TUB6-F | GAAACCTTGAAGACAGTCGCAAT |
| TUB6-R | GCAATCTGGTGCTGGAAACAA |
| ACT2-F | GTCGTACAACCGGTATTGTGC |
| ACT2-R | CACAAACGAGGGCTGGAACAAG |
| STM-P1-F | GGAAGCTGATTGTTGAAGCATA |
| STM-P1-R | TTGTTGGTGGTGTGACTGATAT |
| STM-P2-F | TTCCTTCGTACAATCCTCCA |
| STM-P2-R | TAAGTGATCTAACATT |
| STM-P3-F | AGACGTAGTAATACTAGA |
| STM-P3-R | GTTCTCGACCTTTTTGGT |
| STM-P4-F | AGACGTAGTAATACTAGA |
| STM-P4-R | GAAACACATTAAAGTACTA |
| STM-P5-F | AGACGTAGTAATACTAGA |
| STM-P5-R | CTACTTTGTTGGTGGTGTG |
| STM-a-F | CTTGTCCAATGGCTTTTGC |
| STM-a-R | TTGTTGGTGGTGTGACTGAT |
| STM-b-F | AGGGTTTCCCAATAAATTTA |
| STM-b-R | TATTATTATTCACTTTGGC |
| STM-c-F | TGGTCTCTCTTCTGCTGCT |
| STM-c-R | AAGAGTAAGTAACTGTGTG |
| STM-d-F | CATAAATGAGAAATCATAT |
| STM-d-R | GGAAGATAAATCTAAGGAA |
| STM-e-F | ACATGATAATGGTGGCAAATA |
| STM-e-R | TAATCATAGAGTAAAACAT |
| STM-f-F | CAACATATTTTGGATAACTA |
| STM-f-R | AATAGATAGAGAGAGATTAC |
| STM-g-F | AATAAGTTTGTTTAGTAGAA |
| STM-g-R | TTATTGAAAACGAACGTATAT |
| STM-h-F | TTGTTTTAGAACTTTTTCT |
| STM-h-R | CATTTGCTAAAAGCCCAC |
| STM-i-F | GTTCTTATCTAAGCTATGAAT |
| STM-i-R | TTGACCAAAAAATAACTG |
| STM-j-F | ATATTATTTTTTCACAAGC |
| STM-j-R | ATTCCAAACATTATTGGACT |
| STM-k-F | AGACGTAGTAATACTAGATC |
| STM-k-R | TTTGGATGAAAATGGTTGT |
| AP1-F | ATAATCGTTACGGTTACTAAAGT |
| AP1-R | TGACGTTAGAGCTTAGAAGACC |
